# Analysis of the Taxonomy, Synteny, and Virulence Factors for Soft Rot Pathogen *Pectobacterium aroidearum* in *Amorphophallus konjac* Using Comparative Genomics

**DOI:** 10.3389/fmicb.2022.868709

**Published:** 2022-07-13

**Authors:** Yanan Zhang, Honglong Chu, Liqiong Yu, Fei He, Yong Gao, Lizhou Tang

**Affiliations:** ^1^College of Biological Resource and Food Engineering, Yunnan Engineering Research Center of Fruit Wine, Qujing Normal University, Qujing, China; ^2^School of Modern Agriculture and Biotechnology, Ankang University, Ankang, China; ^3^College of Life Sciences, Jiangxi Normal University, Nanchang, China

**Keywords:** bacterial soft rot, *Pectobacterium aroidearum*, comparative genomics, genomic rearrangement, *Amorphophallus konjac*

## Abstract

Bacterial soft rot is a devastating disease for a wide range of crops, vegetables, and ornamental plants including konjac (*Amorphophallus konjac*). However, the pangenome and genomic plasticity of the konjac soft rot pathogens is little explored. In this study, we reported the complete genome sequences of 11 bacterial isolates that can cause typical soft rot symptoms in konjac by *in vitro* and *in vivo* pathogenicity tests. Based on *in silico* DNA–DNA hybridization, average nucleotide identity and phylogenomic analysis, all 11 isolates were determined to be *Pectobacterium aroidearum*. In addition, synteny analysis of these genomes revealed considerable chromosomal inversions, one of which is triggered by homologous recombination of ribose operon. Pangenome analysis and COG enrichment analysis showed that the pangenome of *P*. *aroidearum* is open and that accessory genes are enriched in replication, recombination, and repair. Variations in type IV secretion system and type VI secretion system were found, while plant cell wall degrading enzymes were conserved. Furthermore, sequence analyses also provided evidence for the presence of a type V secretion system in *Pectobacterium*. These findings advance our understanding of the pathogenicity determinants, genomic plasticity, and evolution of *P*. *aroidearum*.

## Introduction

Bacterial soft rot is a disease of agricultural ecosystems, caused by multiple genera of Gram-negative and Gram-positive bacteria including *Pseudomonas*, *Bacillus*, *Burkholderia*, *Pantoea*, *Enterobacter*, *Klebsiella*, *Leuconostoc* and *Clostridium* (Charkowski, 2018). Soft rot Pectobacteriaceae (SRP), belonging to the genera *Pectobacterium* and *Dickeya*, are the most widely studied soft rot bacterial pathogens. They infect a broad range of important crop and ornamental plants, leading to economic and yield losses in the field and in storage (Toth et al., 2021a). Konjac (*A. konjac*), a perennial plant belonging to the family *Araceae*, is widely grown as a cash crop in tropical and subtropical Asian countries such as China, India, and Japan. The konjac glucomannan (KGM), extracted from the corm, is a water-soluble polysaccharide (dietary fiber) with diverse applications in food science and nutrition, biotechnology, pharmacology and fine chemicals (Behera and Ray, 2016; Zhu, 2018). However, bacterial sot rot is becoming a major threat to konjac production in China (Wu et al., 2015).

Based on 16S rRNA gene sequence analysis and biochemical tests, the causal agent of konjac soft rot was identified as *Pectobacterium aroidearum* in the Yunnan province of southwestern China (Wei et al., 2020). In recent years, *P*. *aroidearum* was also reported to cause soft rot in Chinese cabbage (Xie et al., 2018), *Cucurbita pepo* (Moraes et al., 2017), *Syngonium podophyllum* (Xu et al., 2020) and Olecranon Honey Peach (Liang et al., 2022). It should be noted that *P*. *aroidearum* was not proposed as a novel species until 2013 (Nabhan et al., 2013). Earlier studies identified *P. carotovorum* subsp. *carotovorum* and *P. chrysanthemi* as the soft rot pathogen of Konjac (Wu et al., 2011). In general, the taxonomy of SRP has been in a state of flux especially over the last two decades due to the development of genome-based taxonomic tools (Toth et al., 2021b). According to the List of Prokaryotic names with Standing in Nomenclature (LPSN), the genus *Pectobacterium* has 19 child taxa with a validly published and correct name (Parte et al., 2020).

Advances in DNA sequencing technology have made large volumes of genomic data available (Land et al., 2015). The use of genome data promises increasing precision and accuracy for the taxonomy of prokaryotes especially at species and subspecies levels (Maiden et al., 2013; Chun et al., 2018). Based on average nucleotide identity (ANI), *in silico* DNA–DNA hybridization (*is*DDH) and phylogenomic analysis, new studies have illustrated inconsistencies between established taxonomies and evidence from completely sequenced isolates such as subspecies of *P. carotovorum* (Pritchard et al., 2016; Zhang et al., 2016). Given the importance of correct taxonomy and the increasing availability of whole-genome sequences, using genomic data will likely become routine for microbial taxonomy in the near future (Meier-Kolthoff et al., 2021).

Comparative genomics, including pangenome analysis, has also been used to reveal the basis of pathogenicity, genomic diversity, pathogenic evolution and host adaptation (Toth et al., 2006; Sheppard et al., 2018; Amir et al., 2020). For *Pectobacterium* spp., genome-wide analyses have indicated considerable variation in the pathogenicity determinants including phytotoxins, polysaccharides, iron uptake systems, asecretion systems (type IV-VI), antimicrobial compounds, and CRISPR-Cas systems, whereas the plant cell wall degrading enzymes (PCWDEs) are highly conserved (Li et al., 2018, 2019; Arizala and Arif, 2019). Of particular interest to soft rot, pangenome analyses have also been performed for some species of *Pectobacterium* such as *P*. *actinidiae* (Lu et al., 2021) and *P. parmentieri* (Zoledowska et al., 2018).

The present study aims to further investigate the causal agent of konjac soft rot and determine its taxonomy based on genomic data. We achieved this by isolating the soft rot pathogens of konjac and assembling 11 complete genomes using Nanopore and Illumina sequencing. From this, we performed comparative genomics and pangenome-oriented analyses for *Pectobacterium* spp. and *P*. *aroidearum* strains. Overall, the obtained results provide new insights into the pathogenicity determinants, genomic structure and evolution of *P*. *aroidearum*.

## Materials and Methods

### Sample Collection and Isolation of Bacterial Strains

Konjac corms with symptoms of soft rot were collected from Qujing City, Yunnan Province and Ankang City, Shaanxi Province, China in 2019 and 2020 (Supplementary Table 1). Infected tissues were cut into small pieces and were sterilized in 75% (v/v) ethanol for 30 s, followed by three times rinses with sterile distilled water. The tissues were then mashed and diluted in sterile distilled water. A volume of 200 μl bacterial suspension from each dilution was spread on nutrient agar (NA) medium and incubated at 28°C for 48 h. A single colony was then picked and subcultured in nutrient broth (NB) medium. Pure cultures were obtained through successive streaking on NA.

### Pathogenicity Tests

For konjac slice assay, bacterial strains were grown overnight, washed and resuspended into sterile water with an OD_600_ = 0.2. The tubers were sliced 0.5 cm thick and placed in a plastic food container containing wet paper tissues. Tuber slices were then inoculated with 20 μl of bacterial solution and incubated at 28°C for 48 h. Sterile water was used as a negative control. For *in vivo* assay, bacterial suspension was prepared with a concentration as mentioned above. Stems of 6-months-old konjac seedlings were then inoculated using a pin-prick inoculation method under greenhouse conditions. Similarly, sterile water was used as a negative control. The assay was repeated twice independently.

Pectinolytic activity assay for bacterial isolates was performed on crystal violet pectate (CVP) medium (Hélias et al., 2012). Briefly, pure bacterial suspensions were diluted into 10^2^–10^3^ CFU/ml. A volume of 100 μl dilution was then plated on a CVP medium and incubated at 28°C for 48 h.

### Genome Sequencing, *de novo* Assembly, and Annotation

Eleven pathogenic strains (per isolate per sample) were selected and then sequenced. Genomic DNA extraction, sequence library construction and sequencing were conducted at the Beijing Novogene Bioinformatics Technology Co., Ltd. Briefly, total DNA from each isolate at the exponential stage was extracted and assessed using agarose gel electrophoresis and Qubit 2.0 Fluorometer (Thermo Scientific). After library construction, sequencing was performed using a Nanopore PromethION platform and an Illumina NovaSeq PE150 platform. For most bacterial isolates, a hybrid assembly pipeline was conducted by Unicycler v0.4.8 using both Illumina reads and long reads (Wick et al., 2017). The genome of strain QJ003 was assembled by Raven v1.5.1 (Vaser and Šikić, 2021) and further polished by Pilon v1.24 (Walker et al., 2014). The quality of all genome assemblies was assessed by BUSCO v5.2.2 based on the dataset bacteria_odb10 (Manni et al., 2021). Prokka v1.14.5 was used for genome annotation (Seemann, 2014).

### Average Nucleotide Identity and Digital DNA–DNA Hybridization Analyses

Pairwise average nucleotide identity (ANI) values were calculated by a Python module pyani v0.2.11 using ANIm method.^1^ *is*DDH was estimated *via* the Genome-to-Genome Distance Calculator 3.0 web server using the recommended formula 2^2^ (Meier-Kolthoff et al., 2021).

### Comparative Genomic Analyses

In addition to our 11 new assemblies, 53 complete genome sequences of *Pectobacterium* were retrieved from GenBank in May 2021.^3^ The accession numbers and other basic information for these downloaded genomes are provided (Supplementary Table 2). After confirming that all 11 *P. aroidearum* assembly sequences began with the gene *dnaA*, bacterial synteny was evaluated and visualized by multiple whole-genome alignment using progressiveMauve (Darling et al., 2010). Dot plot between two genomes was generated by D-Genies web server^4^ (Cabanettes and Klopp, 2018). A comparative genomic ring plot was generated using BLAST Ring Image Generator (BRIG; Alikhan et al., 2011).

### Phylogenetic Analyses

Parsimony trees were inferred with kSNP v3.1.2 based on pan-genome SNPs (Gardner et al., 2015). The optimum k-mer size used for the *Pectobacterium* species and *P*. *aroidearum* isolates was 21 and 19, respectively, which was determined by subcommand Kchooser.

A maximum-likelihood (ML) tree for the *P*. *aroidearum* strains was inferred by FastTree 2.1.11 using generalized time-reversible (GTR) models based on core-gene alignment (Price et al., 2010). The Newick tree files were visualized using the online program iTOL v5 (Letunic and Bork, 2021) and MEGA11 (Tamura et al., 2021).

### Pangenome Analyses and Functional Enrichment

The Roary pipeline was used to infer the pangenome and a gene presence/absence matrix of *Pectobacterium* spp. and *P*. *aroidearum,* respectively (Page et al., 2015). The parameters for minimum blastp percentage identity (−i) and core gene (−cd) were adjusted to 90% and 100%, respectively.

For enrichment analysis, a COG functional category was assigned to the protein-coding genes of *P*. *aroidearum* QJ036 using eggNOG-mapper v2.1.4 (Cantalapiedra et al., 2021). Functional enrichment analysis was performed using Fisher’s exact test within the R environment (v4.0.0). *p* values for multiple comparisons were adjusted with the Benjamini and Hochberg method (Benjamini and Hochberg, 1995).

### Identification of Virulence Factors

Protein secretion systems (types I, II, III, IV, V, and VI) were detected by uploading the protein sequences to TXSScan/MacSyFinder (Abby et al., 2014, 2016). SecRet6^5^ was used to predict the T6SS-associated proteins (Li et al., 2015). Domain identification and protein family classification was conducted by InterProScan (Blum et al., 2021). Multiple sequence alignment was then performed by T-Coffee (Di Tommaso et al., 2011) and visualized by ENDscript (Robert and Gouet, 2014).

PCWDE proteins of the genera *Pectobacterium* and *Dickeya* were downloaded from the UniProt database and used for building a reference database. BLAST+ (v2.6.0) was applied to find putative PCWDEs in 14 *P*. *aroidearum* genomes using an E-value threshold (E-value < 1e − 5). Only hits with at least 35% identity and 50% query coverage were kept. If a protein annotation from BLAST+ results was not consistent with that derived from eggNOG-mapper (v2.1.4), this protein annotation was examined manually.

Genomic islands (GIs) were predicted using the IslandViewer 4 webserver.^6^ IslandViewer 4 integrated three GI prediction methods (IslandPath-DIMOB, SIGI-HMM and IslandPick) as well as annotations of virulence factors, pathogen-associated genes, and antimicrobial resistance (AMR) genes.

## Results

### Determination of the Causal Agent of Konjac Soft Rot

**Figure 1:**
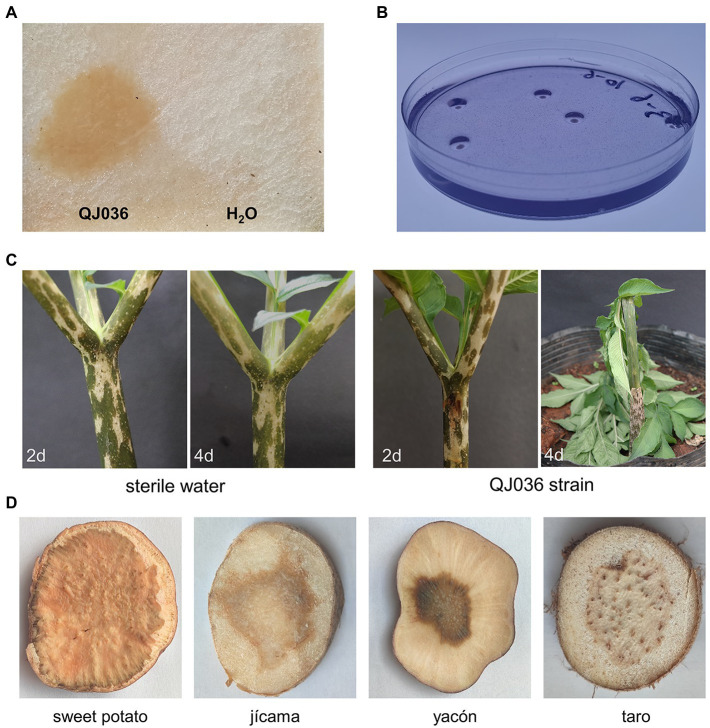
Pathogenicity tests and host range determination of *Pectobacterium aroidearum* QJ036. **(A)** Bacterial cultures of QJ036 strain (10^8^ CFU/ml) were inoculated on konjac slice tubers for 24 h. Sterile water was also used as negative control. **(B)** The formation of pits caused by QJ036 strain on CVP medium incubated at 28°C for 48 h. **(C)** Bacterial cultures of QJ036 strain (10^8^ CFU/ml) and sterile water were inoculated into stems of 6-months-old konjac seedlings, respectively. Pictures were taken at the indicated time points. **(D)** Bacterial cultures of QJ036 strain (10^8^ CFU/ml) were inoculated on slice tubers of indicated species for 24 h. All these experiments were repeated at least twice independently with similar results.

To further determine the causal agent of konjac soft rot in China, *A. konjac* tubers with foul-smelling rot symptoms were collected from Qujing, Yunnan Province and Ankang, Shaanxi Province, China. After bacterial isolation and purification, a total of 11 isolates were shown to cause typical soft rot symptoms on konjac tubers *in vitro* (Figure 1A; Supplementary Figure 1). As expected, the pectolytic ability of these candidate pathogens was further confirmed based on the formation of deep cavities on crystal violet pectate (CVP) media (Figure 1B; Supplementary Figure 2). 16S rRNA gene sequencing and BLAST analysis showed that all 11 16S rRNA genes share at least 99% identity with that of *P. aroidearum* (NR_159926), which is consistent with previous findings (Xie et al., 2020). Since all candidate pathogens belonged to the same species based on 16S rRNA gene sequences and morphological similarity, only one strain OJ036 was selected for pathogenicity test *in vivo*. Stem rot and wilting symptoms were visible only within 2 days for seedlings inoculated with strain QJ036 while no symptoms were observed for the control group (Figure 1C). This represents strong evidence that the causal agent of konjac soft rot is *P. aroidearum*.

In addition, we did pathogenicity tests *in vitro* to explore the host range of *P. aroidearum*. Interestingly, *P*. *aroidearum* was found to cause typical rot symptoms on sweet potato (*Ipomoea batatas* (L.) Lam.), jícama (*Pachyrhizus erosus* (L.) Urb.), yacón (*Smallanthus sonchifolius* (Poepp.) H.Rob.) and taro (*Colocasia esculenta* (L.) Schott), which has not been reported (Figure 1D). Although *in vivo* pathogenicity tests are needed, these results suggest a broader host range of *P*. *aroidearum*.

### Phylogenomic Analysis and Genomic Features of *Pectobacterium aroidearum* Strains

**Figure 2:**
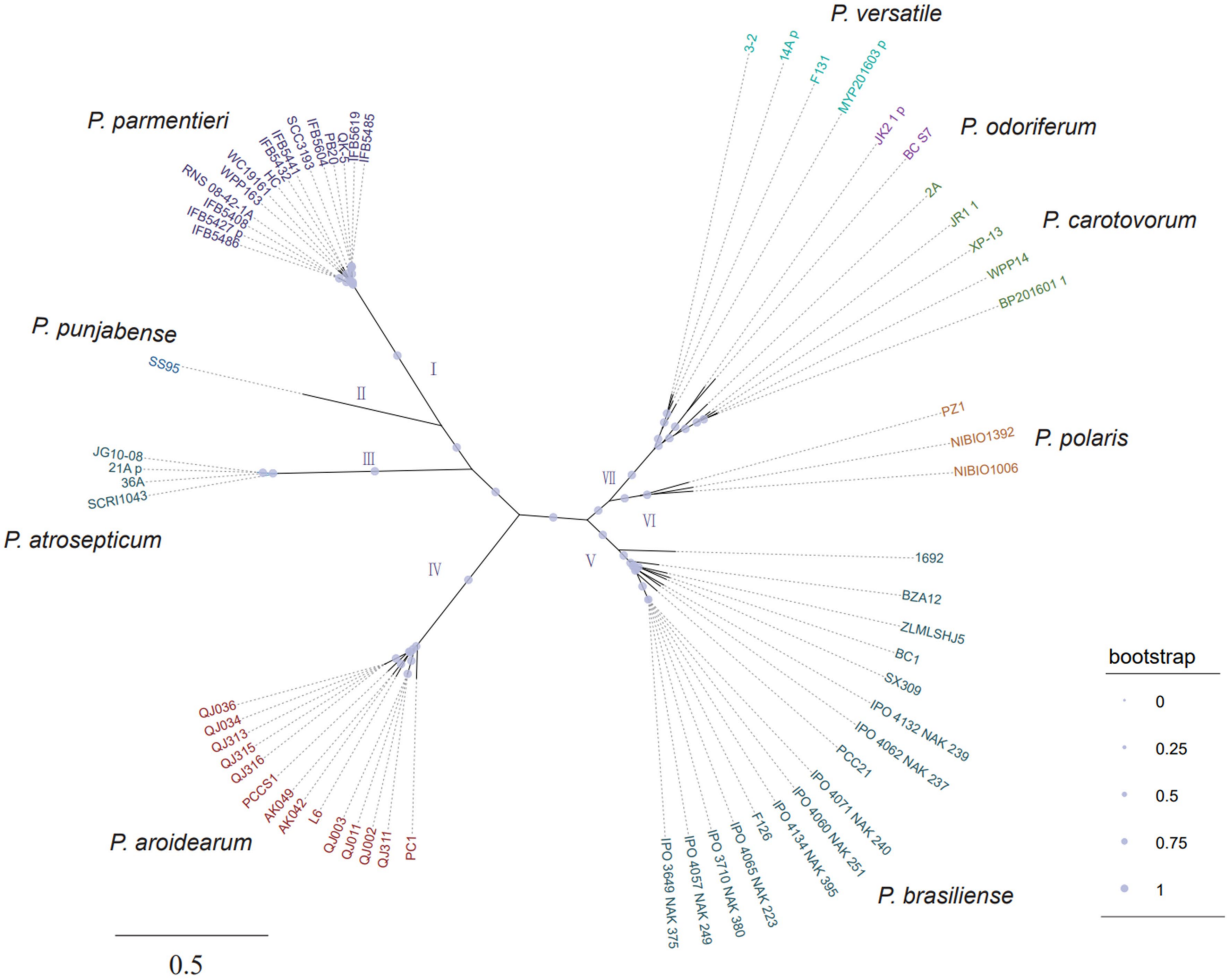
The unrooted parsimony tree of 64 *Pectobacterium* strains based on all SNPs. The consensus parsimony tree was constructed by kSNP3 and visualized by iTOL. The support values were calculated by FastTreeMP. Branch lengths are expressed in terms of changes per number of SNPs.

To further confirm the taxonomic status and investigate the genomic diversity of konjac soft rot pathogens, whole-genome sequencing was performed for the 11 strains using a Nanopore PromethION platform and an Illumina NovaSeq platform with more than 100x coverage depth (Supplementary Table 3). After hybrid genome assembly using filtered data, one circular chromosome without gaps was obtained for each isolate. High scores (>99%) were achieved from a BUSCO assessment of genomic completeness, indicating the high quality of our assemblies (Supplementary Figure 3). The parsimony tree based on genome-wide single nucleotide polymorphisms (SNPs) clustered these *Pectobacterium* spp. into seven well-resolved clades, of which clade VII can be further clustered into three subclades (Figure 2). Notably, all konjac soft rot pathogens form a monophyletic group (clade IV) with *P. aroidearum* L6, *P. carotovorum* subsp. *carotovorum* PC1 and *P. carotovorum* subsp. *carotovorum* PCCS1, indicating that these strains belong to the same species (Figure 2). This result is further supported by average nucleotide identity (ANI) and digital DNA–DNA hybridization (dDDH) analyses, two widely used methods for the taxonomy of prokaryotes (Chun et al., 2018). Sequence comparisons between the trains in clade IV show that ANI is greater than 95% and dDDH greater than 70%, which are above the cut-off values for species delineation (Supplementary Table 4). Taken together, these results clearly indicate that all these strains in clade IV should be classified as *P. aroidearum* and that three strains (*P. carotovorum* subsp. *carotovorum* PC1, *P. carotovorum* subsp. *carotovorum* PCCS1 and *P. carotovorum* subsp. *carotovorum* PCC21) are incorrectly named.

Overall genomic features of *P*. *aroidearum* strains, including genome size, GC (guanine-cytosine) content, number of protein-coding sequences (CDS), and number of RNA genes, were quantified (Table 1). The length of *P*. *aroidearum* genomes ranges from 4,865,541 bp (QJ315) to 5,057,072 bp (QJ003). GC content varies between 51.6% and 51.9%. The highest number of putative CDS was observed in AK042 (4,469), whereas the lowest number of CDS was found in QJ311 (4,277). In addition, the number of tRNA, rRNA and CRISPRs (Clustered Regularly Interspaced Short Palindromic Repeats) is the same among our 11 genome assemblies. We found no evidence of plasmids in *P*. *aroidearum.*

**Table 1:** General genome characteristics of sequenced *Pectobacterium aroidearum* strains.

Strain ID	Genome size	Contig	GC%	CDS	rRNA	tRNA	ncRNA	CRISPR	Plasmid
QJ002	4,975,218	1	51.9	4,361	22	77	98	1	no
QJ003	5,057,072	1	51.9	4,467	22	77	98	1	no
QJ011	5,044,175	1	51.9	4,449	22	77	98	1	no
QJ034	4,889,365	1	51.6	4,308	22	77	98	1	no
QJ036	4,889,381	1	51.6	4,304	22	77	98	1	no
QJ311	4,907,098	1	51.9	4,277	22	77	98	1	no
QJ313	4,889,381	1	51.6	4,304	22	77	98	1	no
QJ315	4,865,541	1	51.7	4,280	22	77	98	1	no
QJ316	4,889,381	1	51.9	4,305	22	77	98	1	no
AK042	5,019,255	1	51.6	4,469	22	77	100	1	no
AK049	5,019,088	1	51.6	4,469	22	77	100	1	no

### Synteny Analysis of *Pectobacterium aroidearum* Strains

**Figure 3:**
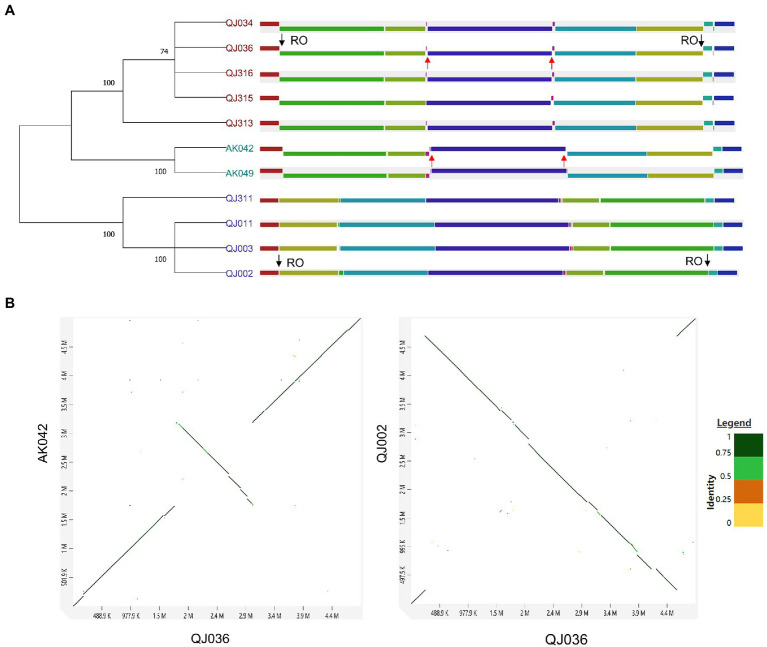
Synteny analysis of *Pectobacterium aroidearum* strains. **(A)** Genome alignment using the progressiveMauve algorithm. Each locally collinear block (LCB) is assigned a unique color. The black arrows indicate the inversion region between QJ036 and QJ002 while the red arrows indicate the inversion region between QJ036 and AK042. RO stands for ribosome operon. SNPs-based parsimony tree of 11 *Pectobacterium* strains was constructed by kSNP3 and the condensed tree with a root at midpoint was computed by MEGA11 with the default cutoff value (≥50). **(B)** Pairwise genome alignment for the selected strains is shown in the dot plots generated by an online tool D-GENIES.

For synteny analysis, the program Mauve was used to perform whole-genome multiple alignment to detect genomic rearrangement. Interestingly, a couple of large-scale chromosomal inversions were observed among these 11 *P*. *aroidearum* isolates (Figure 3A). These strains could be clustered into three groups based on genomic rearrangement, which is further supported by the phylogenetic analysis using genome-wide SNPs (Figure 3A) and BLAST comparisons of the whole genome (Supplementary Figure 4). Except for strain QJ311, the grouping of the other strains is consistent with their geographical distribution (Supplementary Table 1). To further confirm the above results, a total of three strains (QJ002, QJ036 and AK042) selected from each group were used for pairwise genome alignment. As expected, dot plots also revealed the genomic inversions between QJ036 and either QJ002 or AK042 (Figure 3B).

The major mechanisms underlying chromosomal rearrangements are recombinational exchanges between homologous sequences such as ribosomal operons and mobile genetic elements (MEGs) including transposons, insertion sequence (IS) elements, and prophages (Raeside et al., 2014). The origins of the chromosomal inversion were investigated by examining the sequences bordering each inversion. The inversion that occurred between QJ036 and QJ002 resulted from homologous recombination of ribosome operons which are located exactly at the border regions of this inversion (Figure 3A). However, the reason for the inversion between QJ036 and AK042 is unknown.

### Pangenome Analysis and Functional Enrichment of *Pectobacterium* Spp.

The pangenomes of plant pathogens are often associated with species- or strain-specific virulence, host specificity or adaptive potential, or evolutionary history (Amir et al., 2020). To investigate the genomic plasticity of genus *Pectobacterium*, the 64 complete genomes from 9 *Pectobacterium* spp. including our 11 assemblies were used to identify the core- and pan-genomes. The number of core genes was 2,228, which only accounts for 11.31% of the pangenome, which contained a total of 19,698 genes (Supplementary Figure 5A). As more genomes were added, the pangenome trend showed a gradual expansion, implying an open pangenome of *Pectobacterium* strains (Supplementary Figure 5B).

**Figure 4:**
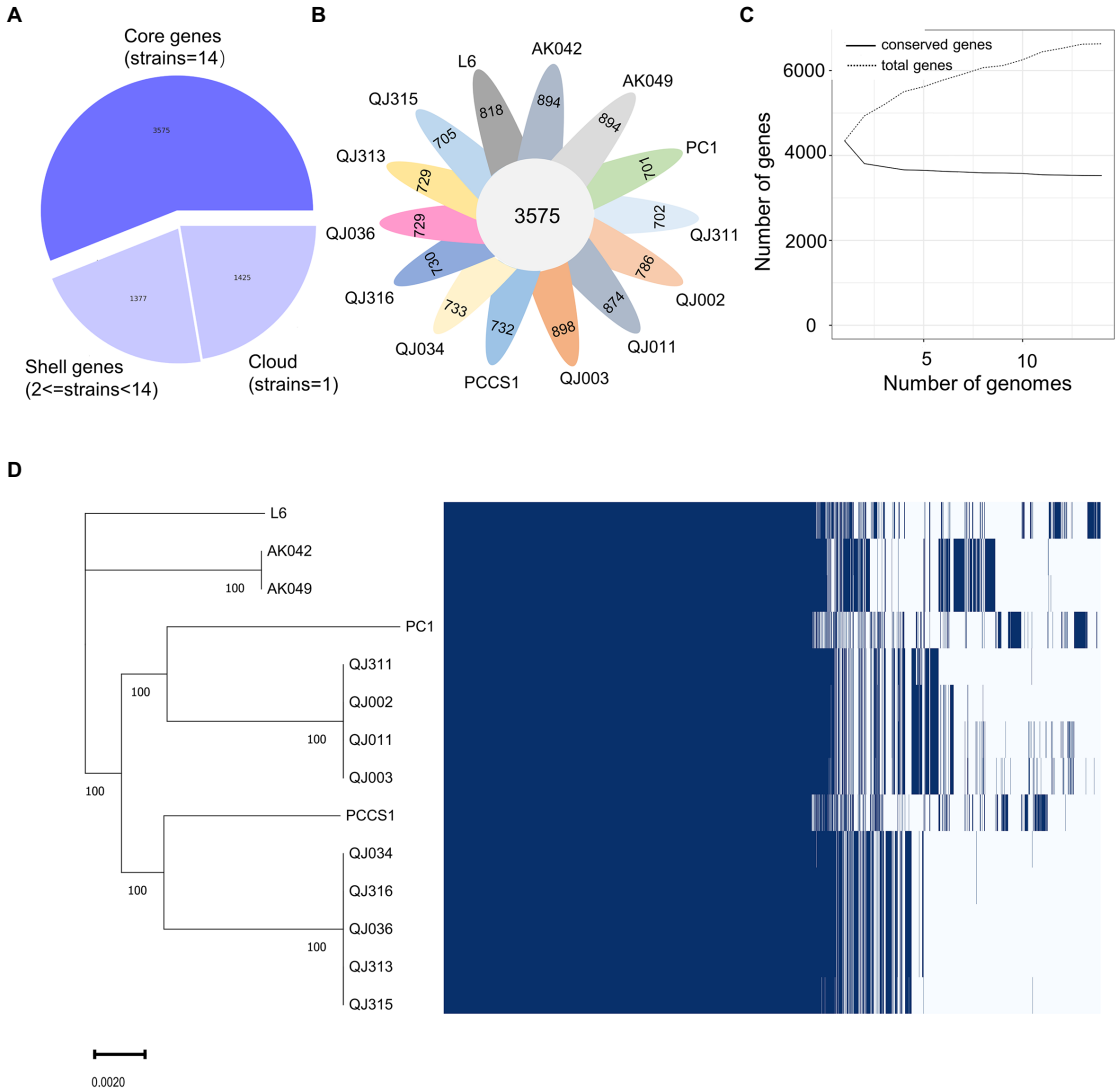
Pangenome analysis of 14 *Pectobacterium aroidearum* strains conducted with the Roary pipeline. **(A)** A pie chart displays the proportion of genes in the core, shell, and cloud of the pangenome. **(B)** A follower plot shows the number of core genes and accessory genes in each *P*. *aroidearum* strain. **(C)** The size of the core genome and pangenome with the increasing numbers of *P*. *aroidearum* genomes. **(D)** Gene presence–absence matrix shows the distribution of genes in each genome. The maximum likelihood (ML) tree is based on all the core gene alignment of the 14 *P*. *aroidearum* genomes. Each column represents an orthologous gene family. Dark blue blocks and light gray indicate the presence or absence of a gene, respectively.

Similarly, a pangenome of 6,630 genes was identified from the 14 *P*. *aroidearum* genomes, with a core genome of 3,575 genes (53.92% of the pangenome; Figure 4A). The number of accessory genes of each strain varied from 701 (PC1) to 898 (QJ003; Figure 4B). The pangenome fitted cumulative curve showed that with the addition of more genomes, the number of core genes remained relatively stable while the number of total genes continued to increase, indicating that the pangenome is still open (Figure 4C). In addition, a maximum likelihood (ML) tree based on core gene alignment is congruent with the matrix representing the presence and absence of core and accessory genes (Figure 4D).

Functional enrichment analysis was performed to uncover the biological roles of core and accessory genes using the QJ036 genome as an example. We classified all genes in the QJ036 genome into three groups including genus-core genes (2228), species-core genes (1347) and accessory genes (729). Enrichment analysis based on the COG (Clusters of Orthologous Groups of proteins) database showed that genus-core genes are overrepresented in categories associated with essential life activity such as energy production and conversion, the transport and metabolism of amino acid, nucleotide and lipid, and translation, ribosomal structure and biogenesis (Table 2). The species-core genes are enriched for virulence-associated categories, including the transport and metabolism of carbohydrate and inorganic ion, cell motility, and intracellular trafficking and secretion (Table 2). Interestingly, the category of replication, recombination and repair is the only enriched COG for the accessory genes (Table 2). Further investigation listed all genes belonging to this category and found that a majority of these genes were annotated as recombinase/integrase and mobile genetic elements such as transposons, insertion sequence (IS) elements (Supplementary Table 5).

**Table 2:** Functional enrichment analyses of genes in *Pectobacterium aroidearum* QJ036.

Functional classification	Abbr.		*P* value	
		Genus-core genes	Species-core genes	Accessory genes
RNA processing and modification	A	1	1	1
Chromatin structure and dynamics	B	1	1	1
Energy production and conversion	C	**2.62E-05**	0.09	3.60E-06
Cell cycle control, mitosis and meiosis	D	0.08	8.05E-03	1
Amino acid transport and metabolism	E	**6.98E-03**	0.91	5.80E-11
Nucleotide transport and metabolism	F	**2.00E-09**	4.46E-03	2.11E-09
Carbohydrate transport and metabolism	G	0.2	**4.46E-03**	4.73E-04
Coenzyme transport and metabolism	H	1.23E-06	0.27	9.82E-13
Lipid transport and metabolism	I	**3.10E-03**	0.41	1.36E-04
Translation, ribosomal structure and biogenesis	J	**2.62E-17**	7.41E-08	2.29E-09
Transcription	K	2.08E-03	0.09	1
Replication, recombination and repair	L	0.05	3.18E-05	**5.20E-11**
Cell wall/membrane biogenesis	M	1	1	1
Cell motility	N	0.14	**0.03**	0.23
Posttranslational modification, protein turnover, chaperones	O	0.08	0.45	0.07
Inorganic ion transport and metabolism	P	0.46	**5.92E-03**	2.78E-14
Secondary metabolites biosynthesis, transport and catabolism	Q	1	0.65	0.23
General function prediction only	R	1	1	1
Function unknown	S	8.28E-04	0.70	0.23
Signal transduction mechanisms	T	1	0.19	2.80E-03
Intracellular trafficking and secretion	U	2.45E-05	**2.25E-04**	1
Defense mechanisms	V	0.01	0.05	1
Extracellular structures	W	1	1	1
Mobilome: prophages, transposons	X	1	1	1
Cytoskeleton	Z	1	1	1

**P*-values were calculated by Fisher exact test and adjusted using Benjamini and Hochberg (BH) method. Over-represented groups (*P*-value < 0.05) were highlighted in bold*.

### Comparison of Key Virulence Factors of *Pectobacterium aroidearum*

**Figure 5:**
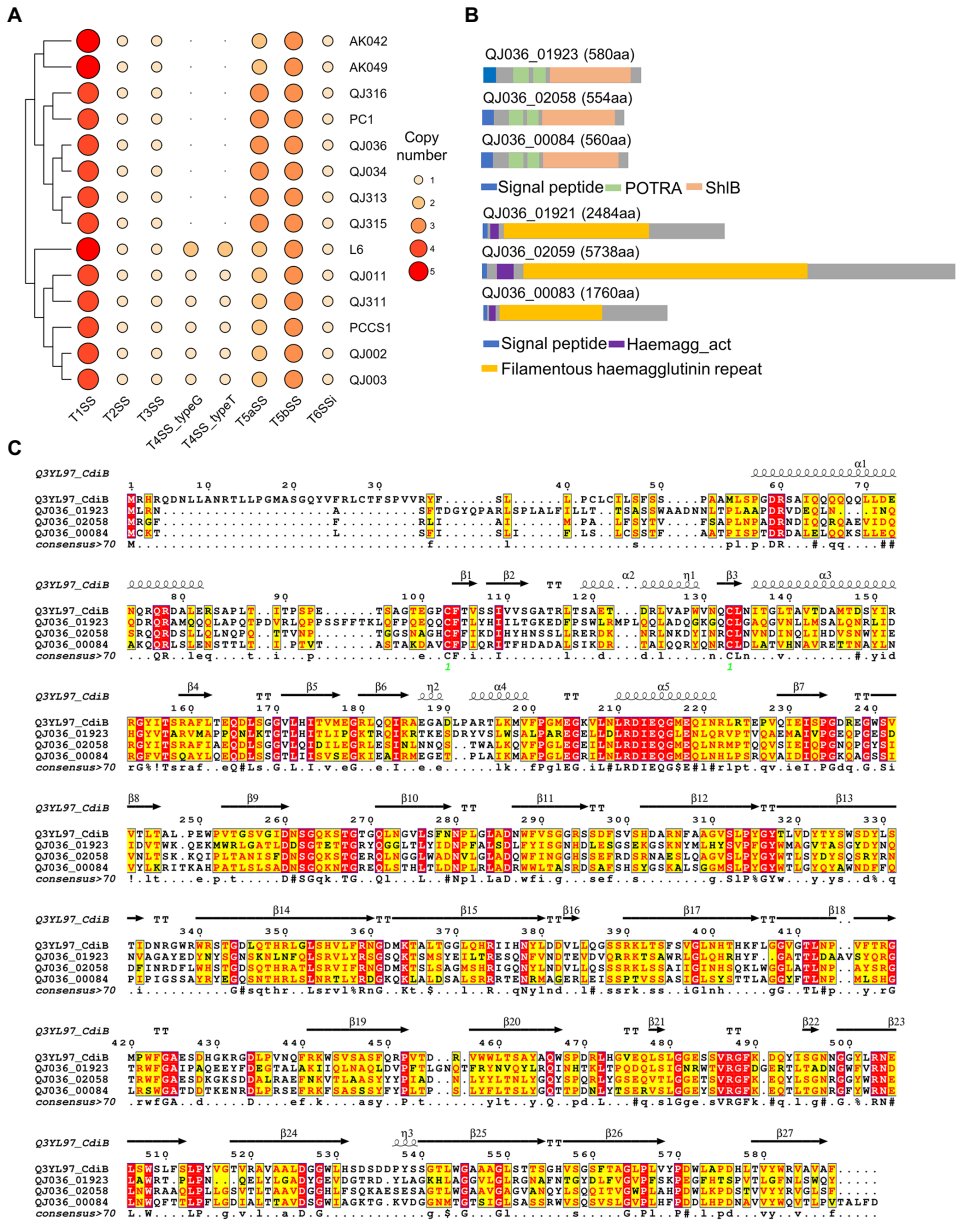
Prediction of bacterial secretion systems in *Pectobacterium aroidearum* strains. **(A)** The heatmap shows the distribution of bacterial secretion systems in *P*. *aroidearum* strains. **(B)** Predicted domains in three putative T5bSS proteins of *P*. *aroidearum* QJ036 using the online software InterProScan. **(C)** Sequence similarities and secondary structure elements from aligned sequences of putative T5bSS proteins and CdiB were rendered by the webserver ESPript 3. The secondary structure depiction is based on CdiB (PDB: 6WIM).

Bacterial secretion systems and plant cell wall degrading enzymes (PCWDEs) play a key role in the interaction between soft rot bacteria and host plants. We mined the 14 *P*. *aroidearum* genomes to identify and compare these determinants of pathogenicity. All six secretion systems (types I–VI) were detected using the program TXSScan. Type I secretion system (T1SS), type II secretion system (T2SS), type III secretion system (T3SS) and type VI secretion system (T6SS) were conserved in all tested *P*. *aroidearum* strains (Figure 5A). However, type IV secretion system (T4SS), a versatile secretion system that is involved in protein translation, bacterial conjugation and DNA uptake/release, is not detected in several *P*. *aroidearum* genomes (Figure 5A). Notably, *P*. *aroidearum* L6 has two copies of each T4SS subtypes (typeG and typeT) that are classified according to different mating pair formation complexes (MPF; Guglielmini et al., 2013). For T6SS, although each *P. aroidearum* strain has a conserved T6SS cluster, the number of predicted effectors and immunity proteins varies considerably (Supplementary Table 6).

Although the presence of type V secretion system (T5SS) in *Pectobacterium* spp. is still inconclusive (Li et al., 2018; Van Gijsegem et al., 2021), two subtypes of T5SS were identified by TXSScan in our assemblies. To further confirm the prediction, sequence analysis was performed using putative T5SS proteins from the QJ036 strain. In two-partner secretion (TPS or type 5b), the passenger domain (TpsA protein) and the β-domain (TpsB protein) are encoded by two separate genes which are frequently, but not always, encoded in an operon (Wells and Henderson, 2013). Domain analysis based on the InterPro database showed that each putative T5bSS protein (QJ036_01923, QJ036_02058 and QJ036_00084) identified by TXSScan contains a signal sequence, two polypeptide-transport-associated (POTRA) domains and a shlB domain (β-domain; Figure 5B). In addition, these proteins displayed high similarity in primary and secondary structure with the known TpsB protein CdiB of *Escherichia coli* (Figure 5C). These results strongly indicate that these three proteins are members of TpsB family. To find the TpsA proteins in *P*. *aroidearum*, TpsA protein fhaB (Uniprot: P12255) of *Bordetella pertussis* was used as a query to search against protein database of QJ036 using BLASTP. Domain analysis of the three BLASTP hits (QJ036_01921, QJ036_02059, and QJ036_00083) shows that each contains a signal sequence, a haemagg_act domain and a filamentous hemagglutinin repeat region (Figure 5B). As expected for a type 5b, these three TpsB proteins are very close to their corresponding TpsA proteins according to the locus tags. These results therefore provide evidence for the existence of three copies of T5bSS in *P*. *aroidearum* QJ036. However, the presence of T5aSS is not well supported by our sequence analysis results.

PCWDEs seem to be very conserved in the *P*. *aroidearum* strains examined in this study. In total, 21 PCWDEs genes were found in each strain including two cellulase genes (*celV*, *celS*), one oligogalacturonate lyase gene (*ogl*), eight pectate lyase genes (*pel1*, *pel2*, *pel3*, *pelX*, *pelY*, *pelW*, *pelE*, *pelL*), two pectin acetylesterase genes (*paeX*, *paeY*), one pectin lyase gene (*pnl*), two pectinesterase genes (*pemA*, *pemB*), three polygalacturonase genes (*pehX*, *pehK*, *pehN*, *pehA*), two rhamnogalacturonan lyase genes (*rhiE*, *rhiN*) and one protease gene (*prtC*; Supplementary Table 7).

### Identification of Genomic Island in *Pectobacterium aroidearum*

**Figure 6:**
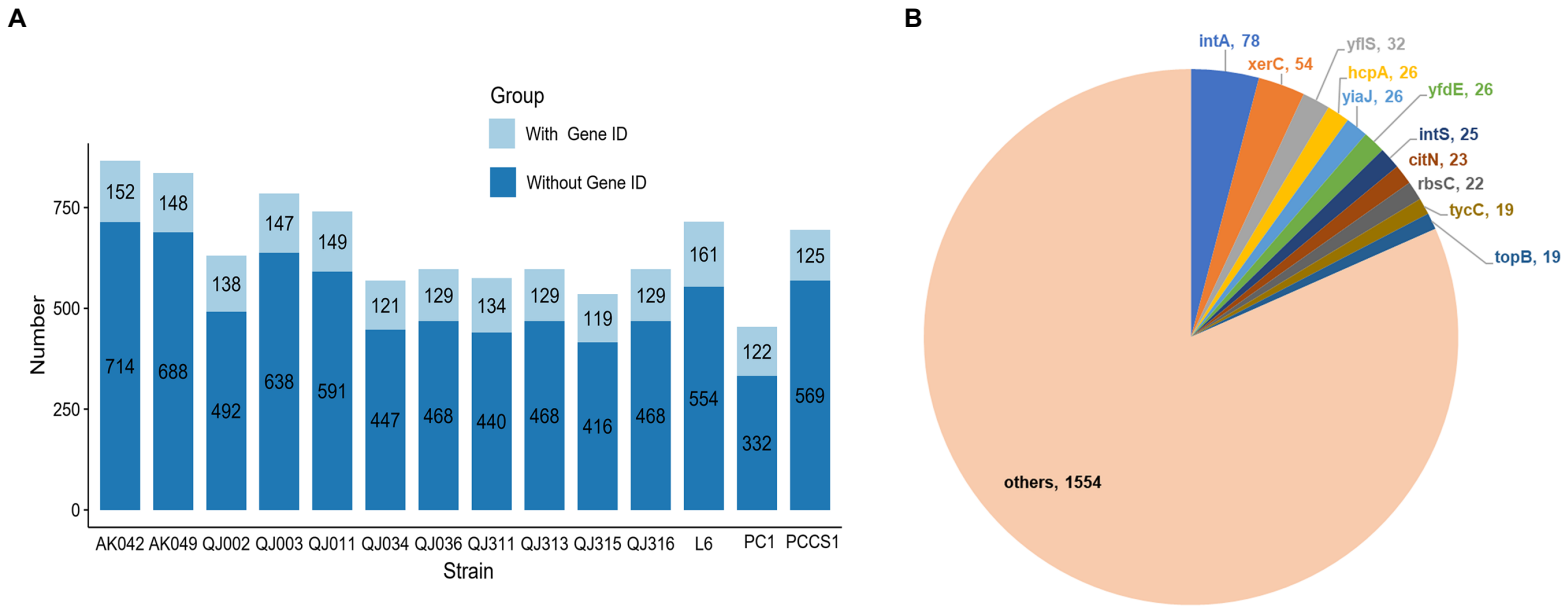
Prediction of genomic islands (GIs) in 14 *Pectobacterium aroidearum* strains. **(A)** The bar plot shows the number of genes from GIs in each *P*. *aroidearum* strain. **(B)** The pie plot shows the frequency of top 11 genes. The values represent the total occurrence number of each gene in 14 *P*. *aroidearum* strains.

Genomic islands (GIs) are clusters of consecutive genes likely acquired *via* horizontal gene transfer (HGT), which may facilitate microbial adaptation by disproportionately encoding factors involved in virulence or antimicrobial resistance (Bertelli et al., 2019). Here, GIs of *P*. *aroidearum* isolates were identified using the online webserver IslandViewer 4 (Bertelli et al., 2017). The location of GIs in each genome was visualized, which revealed that *P*. *aroidearum* isolates with similar GIs distribution patterns tended to be from the same sampling site (Supplementary Figure 6). In addition, the number of GI genes in each genome ranged from 454 (PC1) to 866 (AK042), accounting for about 10%–20% of the total genes of a genome (Figure 6A). However, none of these genes were annotated as virulence/resistance factors, and the majority of these genes were annotated as hypothetical proteins. To further explore the gene content of GIs, we collected all GI-derived genes with a gene ID and calculated the occurrence number of each gene (Supplementary Table 8). The proteins encoded by the top 11 genes include prophage integrase (IntA, IntS), tyrosine recombinase XerC, transporter (YflS, CitN), major exported protein HcpA, DNA-binding transcriptional repressor YiaJ, Acetyl-CoA:oxalate CoA-transferase YfdE, ribose import permease RbsC, Tyrocidine synthase 3 TycC and DNA topoisomerase 3 TopB (Figure 6B).

## Discussion

The identification and classification of the causal agent of konjac soft rot is a prerequisite for effective management of this disease. A nationwide survey in China indicated that konjac soft rot is caused by *P. carotovora* subsp. *carotovora* and *P. chrysanthemi*, with *P. chrysanthemi* acting as the major pathogen (Wu et al., 2015). However, our study isolated 11 pathogenic strains from three sampling sites of two provinces (Yunnan and Shaanxi) in China, and proved that all these soft rot pathogens belong to *P*. *aroidearum*, although it remains possible that konjac soft rot is caused by more than one bacterial pathogen. In addition, considerable variation exists in regard to host specificity for Soft Rot Pectobacteriaceae (SRP; Khadka et al., 2020). Some SRP have wide host ranges, while others have only one or a few plant species (Toth et al., 2021a). *P*. *aroidearum* can infect multiple monocotyledonous and dicotyledonous plant species, especially from the families *Araceae* and *Solanaceae* such as *Zantedeschia aethiopica*, *S. podophyllum*, *A. konjac*, *C. pepo* and *Solanum tuberosum* (Wei et al., 2020; Xu et al., 2020; Toth et al., 2021a). Our *in vitro* pathogenicity tests were the first to show that *P*. *aroidearum* also caused typical rot symptoms on sweet potato, jícama, yacón and taro, suggesting a broader host range of *P*. *aroidearum*. Further research comparing *P*. *aroidearum* to other *Pectobacterium* species with few host plants will help reveal how *P*. *aroidearum* can infect so many plants.

The development of next-generation sequencing technologies (NGS) has made the pangenome a new tool for analyzing pathogenic bacteria (Rouli et al., 2015). To date, there are only a couple of pangenome studies for individual *Pectobacterium* species (Zoledowska et al., 2018; Lu et al., 2021). The core genome size (2,228 genes) of *Pectobacterium* spp. is much smaller than that of *P*. *aroidearum*, and the reduced genes are enriched in virulence-associated COG categories (e.g., the transport and metabolism of carbohydrate and inorganic ion, cell motility, and intracellular trafficking and secretion), which indicates some variation in virulence-related genes among *Pectobacterium* species. As more genomes of *Pectobacterium* species become available, it will be important to directly compare the core genome of each species. Interestingly, our comparative genomic analyses strongly suggest that strains including *P. carotovorum* subsp. *carotovorum* PC1, *P. carotovorum* subsp. *carotovorum* PCCS1 and *P. carotovorum* subsp. *carotovorum* PCC21 are misnamed. Instead, *P. carotovorum* subsp. *carotovorum* PC1 and *P. carotovorum* subsp. *carotovorum* PCCS1 should be classified as *P*. *aroidearum*.

Bacterial genomes are considerably stable in the short term but are plastic from an evolutionary perspective, which creates a delicate balance between genome integrity and instability that is essential for survival and adaptation (Darmon and Leach, 2014). In fact, genomic rearrangements are not only detected across species, but also present in members of the same species for some organisms (Darling et al., 2008). In a long-term evolution experiment using *E. coli*, a total of 110 rearrangement events including 19 inversions were detected, and about 70% of rearrangements were associated with recombination between insertion sequence (IS) elements (Raeside et al., 2014). The importance of IS elements is also highlighted by another study which showed that large inversions were only detected in *Bordetella* species with genomes harboring multicopy IS elements (Weigand et al., 2019). Interestingly, the pangenome analyses and functional enrichment revealed that many accessory genes of *P*. *aroidearum* QJ036 encoded mobile genetic elements, which are likely associated with the dynamics of genome rearrangement. It is known that mobile genetic elements can provide novel genotypes for evolution by facilitating genomic rearrangements and the capture of new genes for bacterial pathogens (Frost et al., 2005; Jackson et al., 2011). Although the effect of these observed chromosomal inversions on fitness and virulence of *P*. *aroidearum* appears to be minor, genome architecture should be taken into consideration when comparing phylogenetically close bacterial pathogens with virulence variation.

The primary virulence determinant of SRP is a large arsenal of plant cell wall-degrading enzymes (Davidsson et al., 2013). Although the number of PCWDE-encoding genes varies slightly across studies, including ours, for the most part these genes seem to be highly conserved (Li et al., 2018, 2019; Arizala and Arif, 2019). Unlike other secreted substrates， T5SS substrates secrete themselves by forming a channel in outer membrane, through which either the remainder of the protein or a separate protein is transported (Green and Mecsas, 2016). For two-partner secretion (T5bSS), the TpsA serves as the secreted protein, which plays an important role in bacterial virulence in *Pseudomonas fluorescens* (Sun et al., 2016) and *B. pertussis* (Melvin et al., 2014).

Here, our sequence analyses provide strong evidence of the existence of three copies of T5bSS in *P*. *aroidearum*. Further research is needed to explore whether these different TpsA proteins, exhibiting substantial size variation, contribute to bacterial pathogenicity. Although it is known that the primary roles of T6SS are associated with both host manipulation and interbacterial competition, how exactly T6SS contributes to virulence is still elusive in *Pectobacterium* (Bernal et al., 2018). In addition, our study suggests that the number of T6SS effectors and immunity proteins varies among *P*. *aroidearum* strains.

Bacterial evolution is dominated by the relative rates of two processes: mutations from DNA replication errors and horizontal gene transfer (HGT; Sheppard et al., 2018). The development of genome sequencing has promoted the realization that HGT is a major evolutionary force reshaping bacterial genomes and therefore influencing bacterial adaptation (Daubin and Szöllősi, 2016). For example, horizontally acquired quorum-sensing regulators expand the host adaptation repertoire in the phytopathogen *P. brasiliense* (Bellieny-Rabelo et al., 2020). Here, IslandViewer 4 was used to find genomic islands including pathogenicity islands (PAIs) in *P*. *aroidearum*. Although 10%–20% of genes appeared in genomic islands, no gene was annotated as a virulence factor, indicating that key pathogenicity determinants of *P*. *aroidearum* are not acquired *via* HGT. For the genes with high frequency such as *intA*, *xerC*, *yflS*, *citN,* further studies are needed to address why these genes are often acquired by HGT as well as their function in *P*. *aroidearum*.

## Data Availability Statement

The raw reads and complete genome assemblies were deposited in the NCBI SRA database and NCBI WGS database, respectively, which is under the bio-project accession number PRJNA794971.

## Author Contributions

YZ and HC designed the experiments and wrote the manuscript. YZ, HC, LY, and FH performed sample collection, experiments, and bioinformatic analysis. YG and LT revised the manuscript. All authors contributed to the article and approved the submitted version.

## Funding

This work was supported by the Special Basic Cooperative Research Programs of Yunnan Provincial Undergraduate Universities (202001BA070001-231) and the National Natural Science Foundation of China (31860057 and 31901468).

## Conflict of Interest

The authors declare that the research was conducted in the absence of any commercial or financial relationships that could be construed as a potential conflict of interest.

## Publisher’s Note

All claims expressed in this article are solely those of the authors and do not necessarily represent those of their affiliated organizations, or those of the publisher, the editors and the reviewers. Any product that may be evaluated in this article, or claim that may be made by its manufacturer, is not guaranteed or endorsed by the publisher.

## Footnotes

^1^ https://github.com/widdowquinn/pyani

^2^ http://ggdc.dsmz.de/ggdc.php#

^3^ https://www.ncbi.nlm.nih.gov/genome/browse/?report=5#!/overview/*Pectobacterium*

^4^ http://dgenies.toulouse.inra.fr/

^5^ http://db-mml.sjtu.edu.cn/SecReT6/

^6^ https://www.pathogenomics.sfu.ca/islandviewer/

## Supplementary Material

Supplementary Figure 1 Pathogenicity tests of other bacterial isolates on konjac slice tuber.

Supplementary Figure 2 The formation of pits caused by other bacterial isolates on CVP medium.

Supplementary Figure 3 BUSCO assessment results of the 11 *P*. *aroidearum* assemblies.

Supplementary Figure 4 Genome comparison of the 11 bacterial isolates using *P*. *aroidearum* QJ002 as the reference by the BLAST Ring Image Generator (BRIG).

Supplementary Figure 5 Pangenome analysis of 64 *Pectobacterium* strains using the Roary pipeline. **(A)** A pie chart displays the proportion of genes in the core, shell, and cloud of the pangenome. **(B)** The size of the core genome and pangenome with the increasing numbers of *Pectobacterium* genomes.

Supplementary Figure 6 Linear visualization of predicted GIs in 14 *P*. *aroidearum* genomes with blocks colored according to the prediction method: IslandPick (green), IslandPath-DIMOB (blue) and SIGI-HMM (orange). Geographic location of these strains are shown on the right side of the figure.

Supplementary Table 1 Basic information of collected isolates *P. aroidearum*.

Supplementary Table 2 Basic information for 53 downloaded genomes of *Pectobacterium* spp.

Supplementary Table 3 Basic statistics of short and long reads.

Supplementary Table 4 Pairwise comparisons of *is*DDH and ANI values of *Pectobacterium* strains in clade IV.

Supplementary Table 5 List of accessory genes that belongs to the category of replication, recombination and repair.

Supplementary Table 6 Type VI secretion system (T6SS) prediction for *P. aroidearum* genomes using web server SecReT6 v3.

Supplementary Table 7 Predicted plant cell wall-degrading enzymes (PCWDEs) in 14 *P. aroidearum* strains.

Supplementary Table 8 The total numbers of each gene derived from GIs.
