# Supplementary figures and images for: Analysis of the Taxonomy, Synteny, and Virulence Factors for Soft Rot Pathogen Pectobacterium aroidearum in Amorphophallus konjac Using Comparative Genomics
Source: Front Microbiol. 2022 Jul 13;13:868709. doi: 10.3389/fmicb.2022.868709 (PMC9326479; doi:10.3389/fmicb.2022.868709)

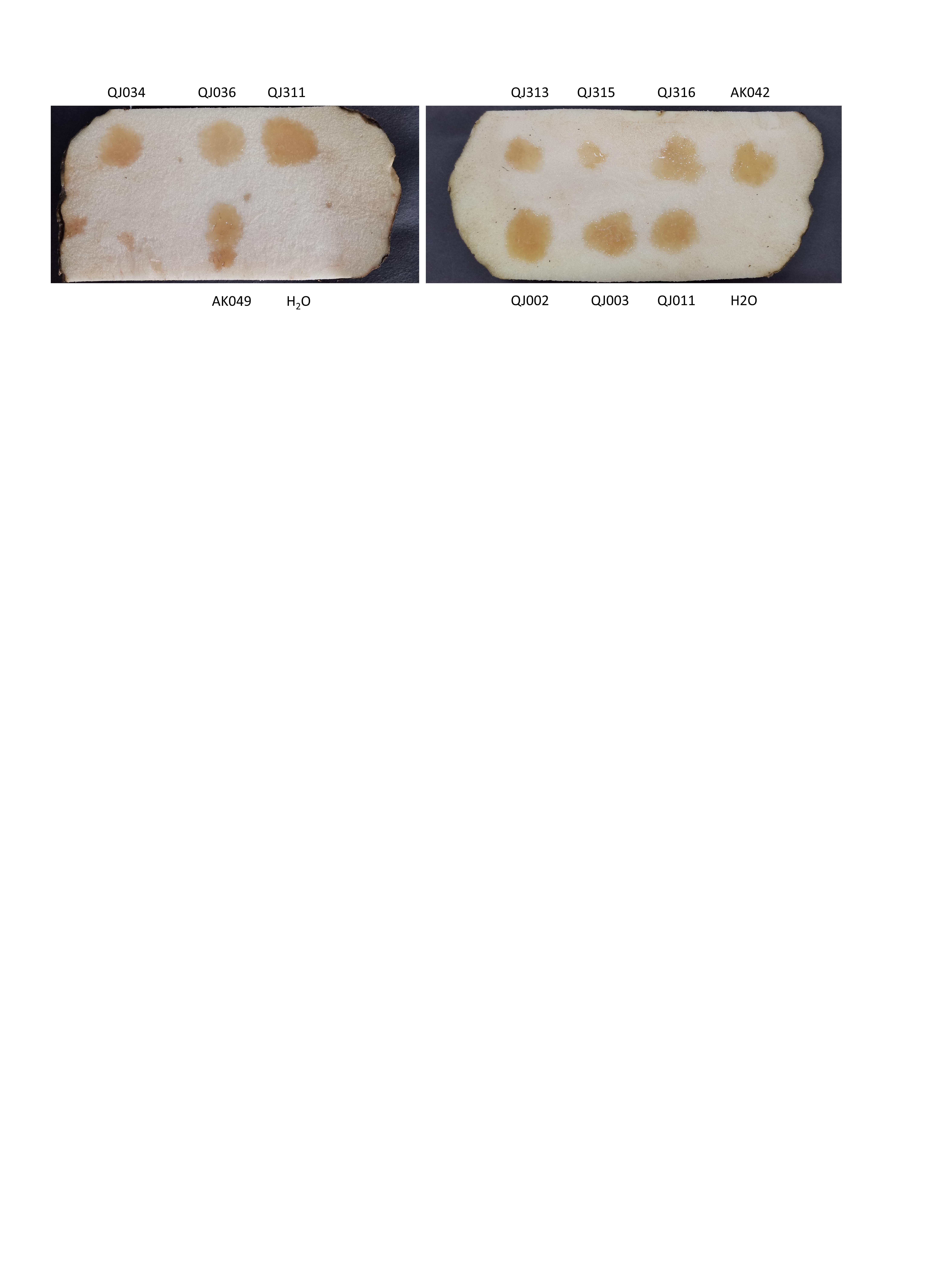

Supplement: Supplementary Figure 1 — Pathogenicity tests of other bacterial isolates on konjac slice tuber. [file Image_1.TIF]

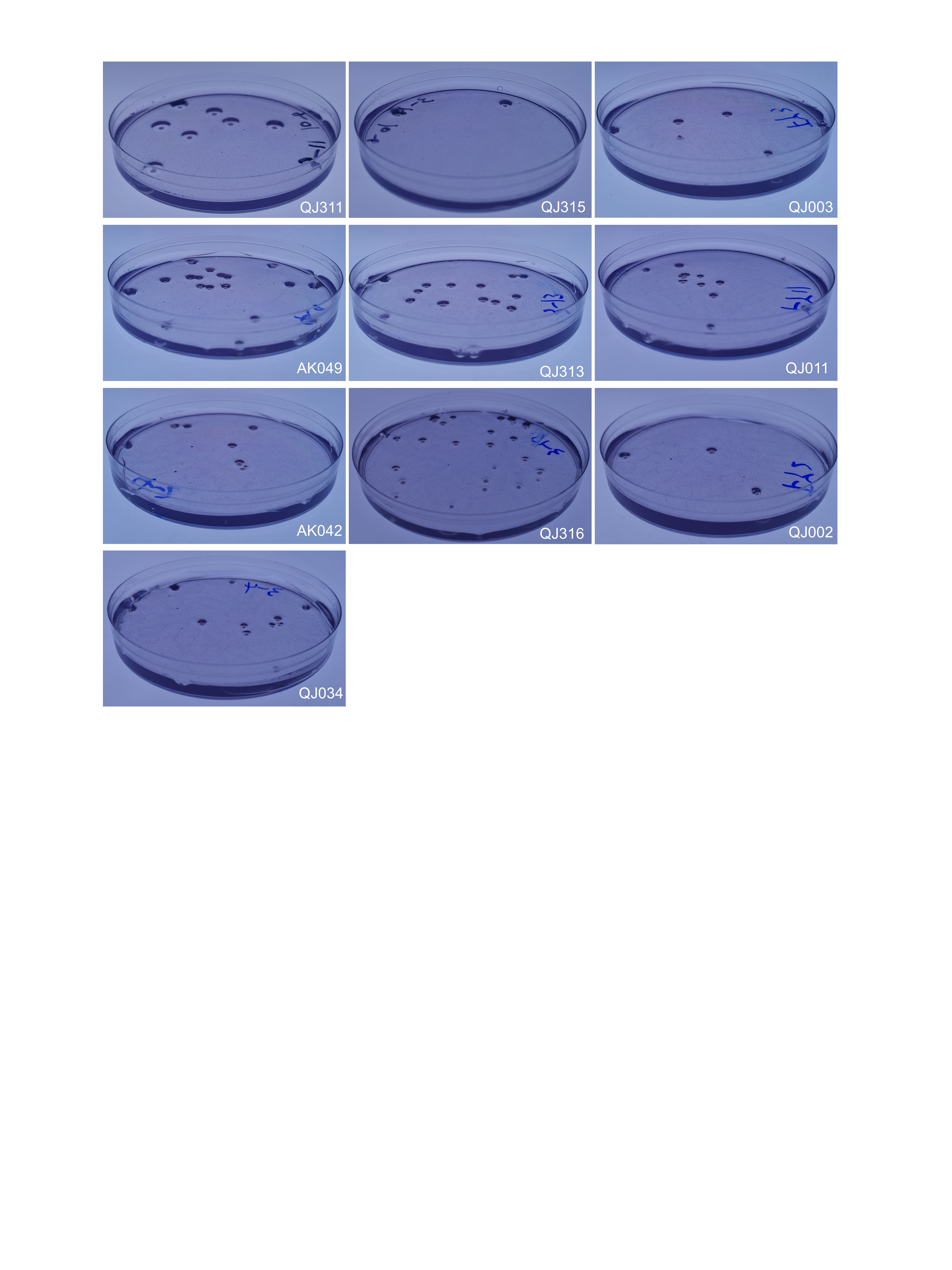

Supplement: Supplementary Figure 2 — The formation of pits caused by other bacterial isolates on CVP medium. [file Image_2.TIF]

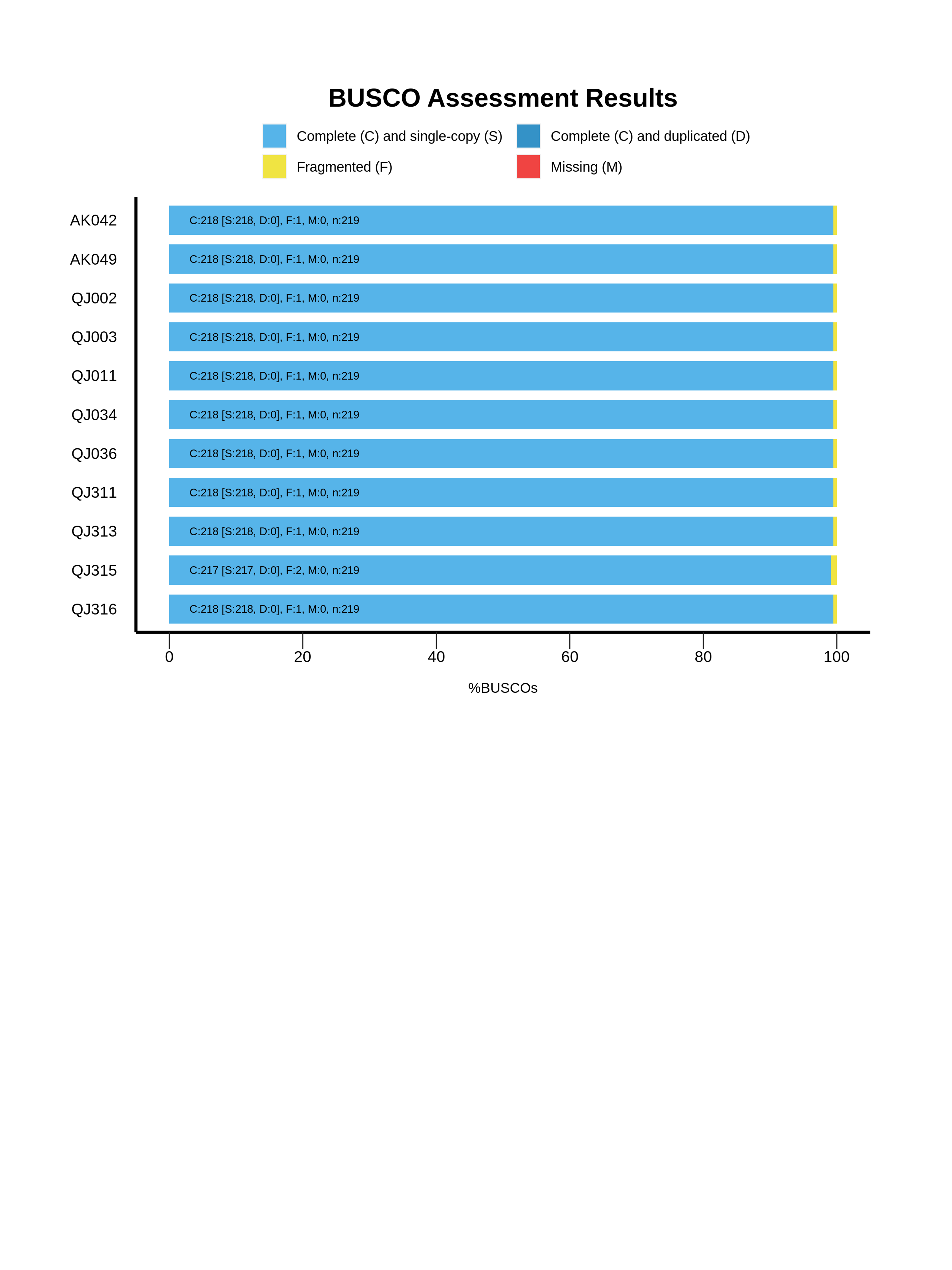

Supplement: Supplementary Figure 3 — BUSCO assessment results of the 11 P. aroidearum assemblies. [file Image_3.TIF]

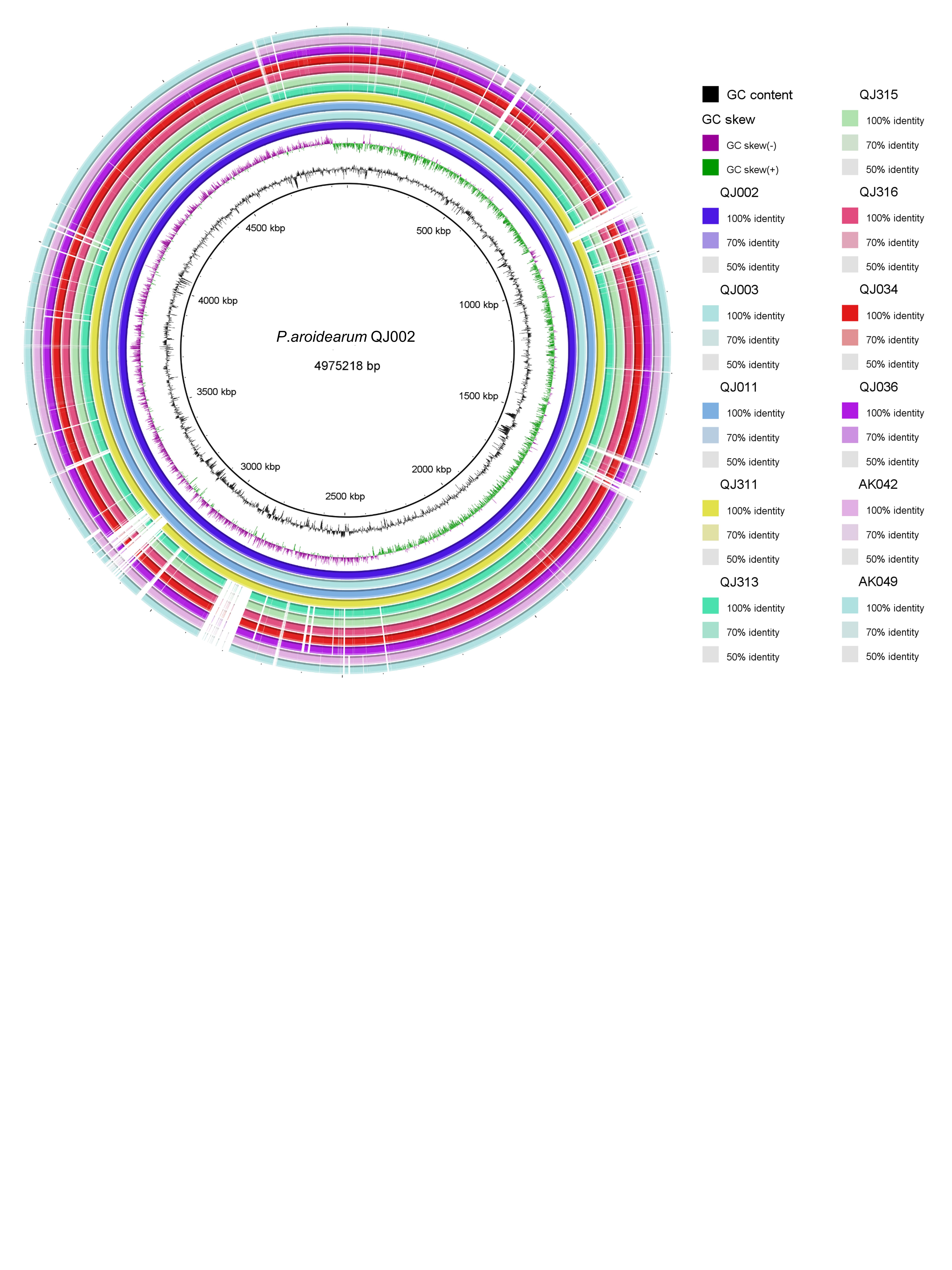

Supplement: Supplementary Figure 4 — Genome comparison of the 11 bacterial isolates using P. aroidearum QJ002 as the reference by the BLAST Ring Image Generator (BRIG). [file Image_4.TIF]

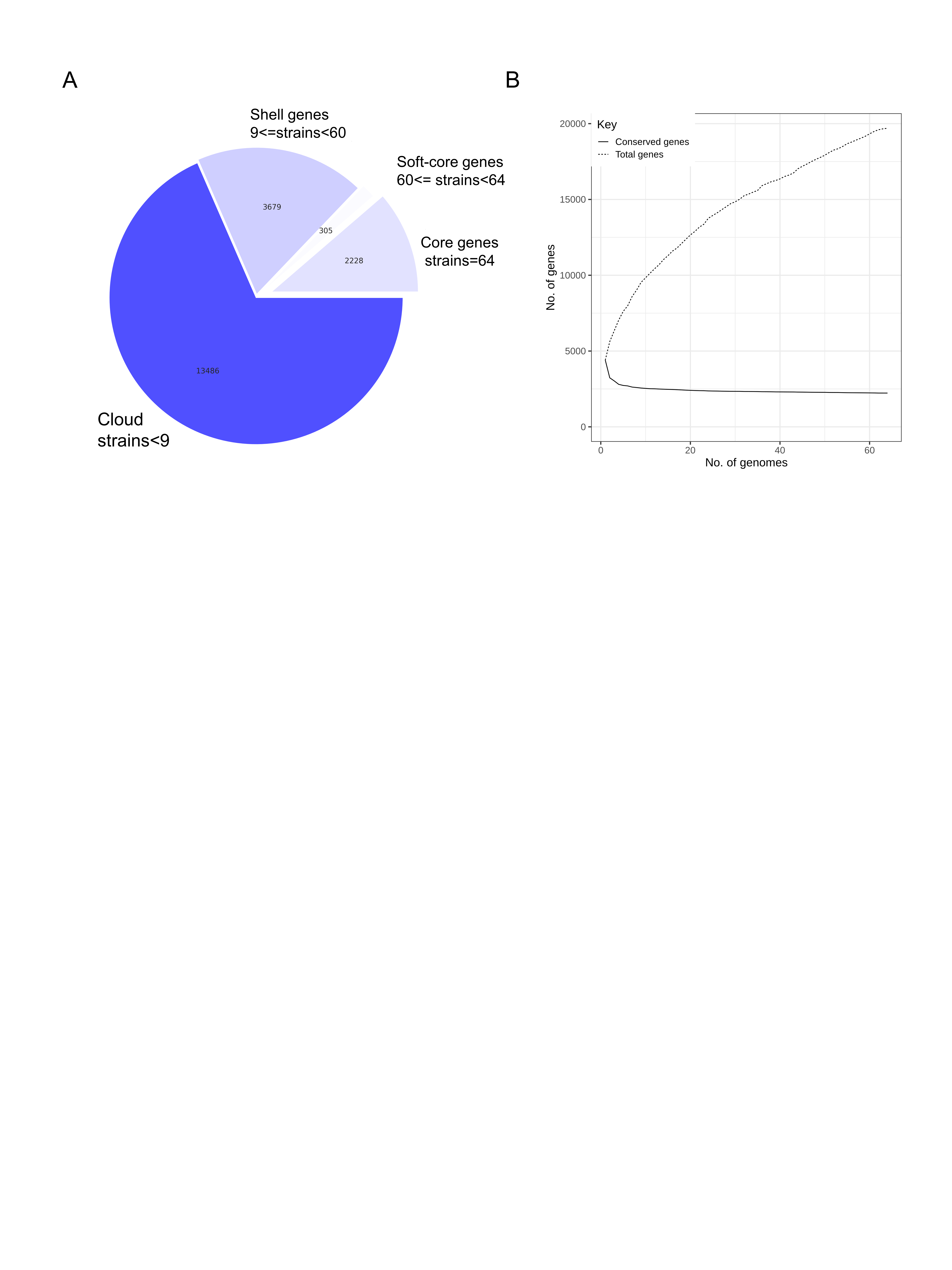

Supplement: Supplementary Figure 5 — Pangenome analysis of 64 Pectobacterium strains using the Roary pipeline. (A) A pie chart displays the proportion of genes in the core, shell, and cloud of the pangenome. (B) The size of the core genome and pangenome with the increasing numbers of Pectobacterium genomes. [file Image_5.TIF]

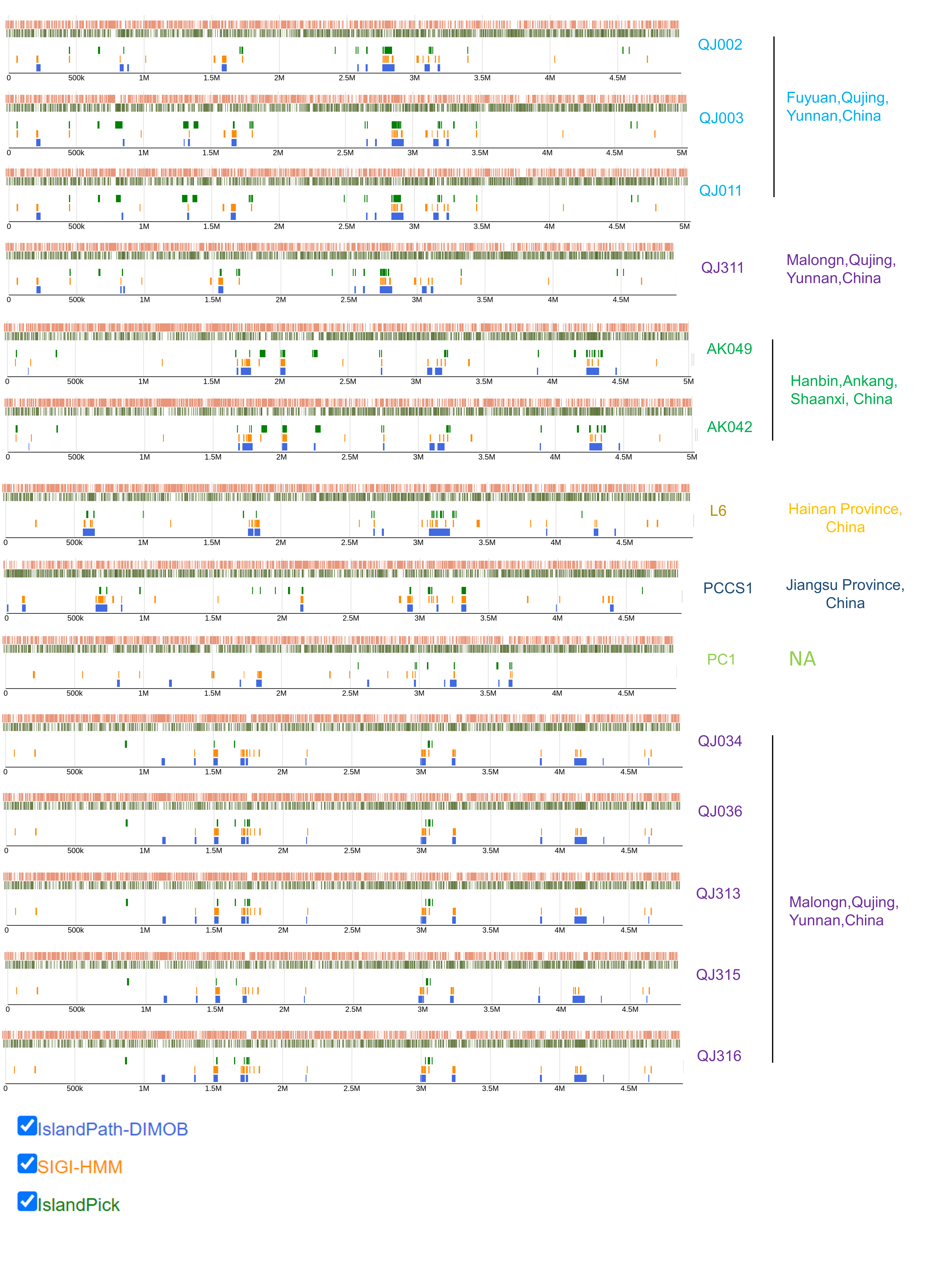

Supplement: Supplementary Figure 6 — Linear visualization of predicted GIs in 14 P. aroidearum genomes with blocks colored according to the prediction method: IslandPick (green), IslandPath-DIMOB (blue) and SIGI-HMM (orange). Geographic location of these strains are shown on the right side of the figure. [file Image_6.TIF]
