# Supplementary material for: Analysis of the Taxonomy, Synteny, and Virulence Factors for Soft Rot Pathogen Pectobacterium aroidearum in Amorphophallus konjac Using Comparative Genomics
Source: Front Microbiol. 2022 Jul 13;13:868709. doi: 10.3389/fmicb.2022.868709 (PMC9326479; doi:10.3389/fmicb.2022.868709)
Supplement: Supplementary Table 1 — Basic information of collected isolates P. aroidearum. [file Table_1.DOCX]

Supplementary Table 1 Basic information of collected isolates *P*. *aroidearum*

| Strain ID | Sampling site | Host | Sampling time |
| --- | --- | --- | --- |
| QJ002 | Fuyuan,Qujing,Yunnan | Amorphophallus konjac | Oct, 2020 |
| QJ003 | Fuyuan,Qujing,Yunnan | Amorphophallus konjac | Oct, 2020 |
| QJ011 | Fuyuan,Qujing,Yunnan | Amorphophallus konjac | Oct, 2020 |
| QJ034 | Malong,Qujing,Yunnan | Amorphophallus konjac | Oct, 2019 |
| QJ036 | Malong,Qujing,Yunnan | Amorphophallus konjac | Oct, 2019 |
| QJ311 | Malong,Qujing,Yunnan | Amorphophallus konjac | Oct, 2019 |
| QJ313 | Malong,Qujing,Yunnan | Amorphophallus konjac | Oct, 2019 |
| QJ315 | Malong,Qujing,Yunnan | Amorphophallus konjac | Oct, 2019 |
| QJ316 | Malong,Qujing,Yunnan | Amorphophallus konjac | Oct, 2019 |
| AK042 | Hanbin,Ankang,Shaanxi | Amorphophallus konjac | Dec,2019 |
| AK049 | Hanbin,Ankang,Shaanxi | Amorphophallus konjac | Dec,2019 |
