# Supplementary material for: Analysis of the Taxonomy, Synteny, and Virulence Factors for Soft Rot Pathogen Pectobacterium aroidearum in Amorphophallus konjac Using Comparative Genomics
Source: Front Microbiol. 2022 Jul 13;13:868709. doi: 10.3389/fmicb.2022.868709 (PMC9326479; doi:10.3389/fmicb.2022.868709)
Supplement: Supplementary Table 3 — Basic statistics of short and long reads. [file Table_3.DOCX]

Supplementary Table 3 Basic statistics of short and long reads

| Sample ID | Illumina | | | | | |  | Nanopore | | | | |
| --- | --- | --- | --- | --- | --- | --- | --- | --- | --- | --- | --- | --- |
|  | Insert Size(bp) | Clean Reads Length(bp) | Raw Data(Mb) | Clean Data(Mb) | Clean Data Q20(%) | Clean Data Q30(%) |  | Number of Reads | Number of Bases(bp) | Mean Read Length(bp) | N50 Read Length(bp) | Mean Read quality |
| QJ036 | 350 | (150:150) | 1637 | 1461 | 97 | 91.59 |  | 69083 | 963655265 | 13949.2 | 15782 | 9.5 |
| QJ315 | 350 | (150:150) | 1837 | 1643 | 96.75 | 91.05 |  | 64688 | 957162962 | 14796.6 | 16748 | 9.6 |
| QJ311 | 350 | (150:150) | 1472 | 1325 | 96.92 | 91.41 |  | 83254 | 1015622172 | 12199.1 | 13495 | 9.6 |
| QJ002 | 350 | (150:150) | 1402 | 1166 | 98.42 | 95.11 |  | 149329 | 1723366317 | 11540.7 | 13843 | 11.7 |
| QJ011 | 350 | (150:150) | 1633 | 1333 | 98.35 | 94.94 |  | 151214 | 1873283580 | 12388.3 | 15060 | 11.7 |
| QJ003 | 350 | (150:150) | 1603 | 1311 | 98.42 | 95.12 |  | 104292 | 1259267737 | 12074.4 | 14439 | 11.7 |
| QJ316 | 350 | (150:150) | 1194 | 1060 | 96.61 | 90.59 |  | 114125 | 1502787643 | 13167.9 | 17182 | 11.6 |
| QJ034 | 350 | (150:150) | 1374 | 1213 | 96.79 | 91.01 |  | 156968 | 1958122029 | 12474.7 | 15963 | 12.1 |
| QJ313 | 350 | (150:150) | 1229 | 1076 | 96.78 | 91.02 |  | 144235 | 1389944535 | 9636.7 | 11763 | 11.6 |
| AK049 | 350 | (150:150) | 1392 | 1198 | 96.88 | 91.26 |  | 271733 | 2352417344 | 8657.1 | 10287 | 11.7 |
| AK042 | 350 | (150:150) | 1189 | 1044 | 97.15 | 91.88 |  | 167222 | 1579425272 | 9445.1 | 11538 | 11.7 |
