# Supplementary material for: Analysis of the Taxonomy, Synteny, and Virulence Factors for Soft Rot Pathogen Pectobacterium aroidearum in Amorphophallus konjac Using Comparative Genomics
Source: Front Microbiol. 2022 Jul 13;13:868709. doi: 10.3389/fmicb.2022.868709 (PMC9326479; doi:10.3389/fmicb.2022.868709)
Supplement: Supplementary Table 4 — Pairwise comparisons of isDDH and ANI values of Pectobacterium strains in clade IV. [file Table_4.DOCX]

Supplementary Table 4 Pairwise comparisons of *is*DDH and ANI values of *Pectobacterium* strains in clade IV

|  | QJ002 | QJ003 | QJ011 | QJ034 | QJ036 | QJ311 | QJ313 | QJ315 | QJ316 | AK042 | AK049 | L6 | PC1 | PCCS1 | *P.polaris* NIBIO1006 |
| --- | --- | --- | --- | --- | --- | --- | --- | --- | --- | --- | --- | --- | --- | --- | --- |
| QJ002 |  | 100.0 | 100.0 | 84.6 | 84.6 | 100.0 | 84.6 | 84.6 | 84.6 | 84.2 | 84.2 | 83.5 | 82.5 | 82.9 | 40.3 |
| QJ003 | 100.00 |  | 100.0 | 84.6 | 84.6 | 100 | 84.6 | 84.6 | 84.6 | 84.2 | 84.2 | 83.5 | 82.4 | 82.9 | 40.3 |
| QJ011 | 100.00 | 100.00 |  | 84.6 | 84.6 | 100 | 84.6 | 84.6 | 84.6 | 84.2 | 84.2 | 83.5 | 82.5 | 82.9 | 40.3 |
| QJ034 | 100.00 | 98.35 | 98.34 |  | 100.0 | 84.6 | 100.0 | 100.0 | 100.0 | 84.5 | 84.5 | 85.3 | 82.7 | 84.4 | 40.3 |
| QJ036 | 98.34 | 98.35 | 98.34 | 100.00 |  | 84.6 | 100.0 | 100.0 | 100.0 | 84.5 | 84.5 | 85.3 | 82.7 | 84.4 | 40.3 |
| QJ311 | 100.00 | 100.00 | 100.00 | 98.36 | 98.36 |  | 84.6 | 84.6 | 84.6 | 84.2 | 84.3 | 83.6 | 82.8 | 82.9 | 40.3 |
| QJ313 | 98.34 | 98.35 | 98.34 | 100.00 | 100.00 | 98.36 |  | 100.0 | 100.0 | 84.5 | 84.5 | 85.3 | 82.7 | 84.4 | 40.3 |
| QJ315 | 98.34 | 98.34 | 98.34 | 100.00 | 100.00 | 98.36 | 100.00 |  | 100 | 84.5 | 84.5 | 85.3 | 82.7 | 84.4 | 40.3 |
| QJ316 | 98.34 | 98.35 | 98.34 | 100.00 | 100.00 | 98.36 | 100.00 | 100.00 |  | 84.5 | 84.5 | 85.3 | 82.7 | 84.4 | 40.3 |
| AK042 | 98.32 | 98.32 | 98.32 | 98.38 | 98.38 | 98.34 | 98.38 | 98.38 | 98.38 |  | 100.0 | 84.5 | 82.5 | 84.4 | 40.5 |
| AK049 | 98.32 | 98.32 | 98.32 | 98.38 | 98.38 | 98.34 | 98.38 | 98.38 | 98.38 | 100.00 |  | 84.5 | 82.5 | 84.4 | 40.5 |
| L6 | 98.30 | 98.30 | 98.30 | 100.00 | 98.43 | 98.31 | 98.43 | 98.43 | 98.43 | 98.41 | 98.41 |  | 82.7 | 82.9 | 40.4 |
| PC1 | 98.12 | 98.15 | 98.12 | 98.14 | 98.14 | 98.16 | 98.14 | 98.14 | 98.16 | 98.14 | 98.14 | 98.18 |  | 82.3 | 40.4 |
| PCCS1 | 98.24 | 98.25 | 98.24 | 98.38 | 98.38 | 98.23 | 98.38 | 98.38 | 98.37 | 98.35 | 98.35 | 98.31 | 98.15 |  | 40.4 |
| *P.polaris* NIBIO1006 | 90.50 | 90.51 | 90.51 | 90.52 | 90.52 | 90.50 | 90.52 | 90.52 | 90.52 | 90.58 | 90.58 | 90.55 | 90.52 | 90.59 |  |

The upper triangle displays *is*DDH values (%), and the lower triangle displays ANI values (%).
