# Supplementary material for: Analysis of the Taxonomy, Synteny, and Virulence Factors for Soft Rot Pathogen Pectobacterium aroidearum in Amorphophallus konjac Using Comparative Genomics
Source: Front Microbiol. 2022 Jul 13;13:868709. doi: 10.3389/fmicb.2022.868709 (PMC9326479; doi:10.3389/fmicb.2022.868709)
Supplement: Supplementary Table 5 — List of accessory genes that belongs to the category of replication, recombination and repair. [file Table_5.DOCX]

Supplementary Table 5 List of accessory genes that belongs to the category of replication, recombination and repair

| Locus tag | COG | Description |
| --- | --- | --- |
| QJ036_03892 | L | AAA family ATPase |
| QJ036_03893 | L | AAA family ATPase |
| QJ036_03723 | L | ATP-binding protein |
| QJ036_01042 | L | CHC2 zinc finger domain-containing protein |
| QJ036_01047 | L | CHC2 zinc finger domain-containing protein |
| QJ036_03940 | L | CRISPR-associated endonuclease/helicase Cas3 |
| QJ036_03933 | L | CRISPR-associated endoribonuclease Cas2 |
| QJ036_03481 | L | DEAD/DEAH box helicase |
| QJ036_03482 | L | DEAD/DEAH box helicase |
| QJ036_01393 | L | DNA cytosine methyltransferase |
| QJ036_00014 | L | DNA mismatch endonuclease |
| QJ036_01395 | L | DNA mismatch endonuclease |
| QJ036_03768 | L | DNA topoisomerase 3 |
| QJ036_01604 | L | helicase |
| QJ036_01611 | L | helicase |
| QJ036_01551 | L | H-NS histone family protein |
| QJ036_01568 | L | H-NS histone family protein |
| QJ036_02719 | KL | hypothetical protein |
| QJ036_03715 | L | hypothetical protein |
| QJ036_04224 | L | hypothetical protein |
| QJ036_01576 | L | integrase arm-type DNA-binding domain-containing protein |
| QJ036_01590 | L | integrase arm-type DNA-binding domain-containing protein |
| QJ036_02717 | L | integrase arm-type DNA-binding domain-containing protein |
| QJ036_03365 | L | integrase arm-type DNA-binding domain-containing protein |
| QJ036_03713 | L | integrase domain-containing protein |
| QJ036_03889 | L | integrase domain-containing protein |
| QJ036_00723 | L | IS110 family transposase |
| QJ036_01591 | L | IS200/IS605 family transposase |
| QJ036_01928 | L | IS4 family transposase |
| QJ036_03440 | L | IS4 family transposase |
| QJ036_00876 | L | IS4 family transposase |
| QJ036_01328 | L | IS4 family transposase |
| QJ036_03171 | L | IS4 family transposase |
| QJ036_01602 | L | IS5 family transposase |
| QJ036_02921 | L | IS5 family transposase |
| QJ036_01562 | L | MobA/MobL family protein |
| QJ036_02740 | L | MobA/MobL family protein |
| QJ036_01987 | L | recombinase family protein |
| QJ036_01988 | L | recombinase family protein |
| QJ036_02617 | L | recombinase family protein |
| QJ036_03700 | L | recombinase family protein |
| QJ036_03742 | L | recombinase family protein |
| QJ036_02746 | L | replication/maintenance protein RepL |
| QJ036_03779 | L | Replicative DNA helicase |
| QJ036_01244 | L | RNA-directed DNA polymerase |
| QJ036_03766 | L | Single-stranded DNA-binding protein |
| QJ036_04211 | L | Single-stranded DNA-binding protein |
| QJ036_01371 | L | site-specific integrase |
| QJ036_03691 | L | site-specific integrase |
| QJ036_04197 | L | site-specific integrase |
| QJ036_01043 | L | site-specific tyrosine recombinase |
| QJ036_01048 | L | site-specific tyrosine recombinase |
| QJ036_03703 | LV | ssDNA-binding domain-containing protein |
| QJ036_01592 | L | TatD family hydrolase |
| QJ036_01986 | L | Tn3 family transposase |
| QJ036_03741 | L | Tn3 family transposase |
| QJ036_02596 | L | TOPRIM and DUF927 domain-containing protein |
| QJ036_04210 | L | TOPRIM and DUF927 domain-containing protein |
| QJ036_03934 | L | type I-E CRISPR-associated endonuclease Cas1e |
| QJ036_03937 | L | type I-E CRISPR-associated protein Cas7/Cse4/CasC |
| QJ036_00013 | L | type II restriction endonuclease |
| QJ036_00016 | L | type II restriction endonuclease |
| QJ036_02600 | L | type II toxin-antitoxin system RelB/DinJ family antitoxin |
| QJ036_01372 | L | tyrosine-type recombinase/integrase |
| QJ036_01400 | L | tyrosine-type recombinase/integrase |
| QJ036_02591 | L | tyrosine-type recombinase/integrase |
| QJ036_03479 | L | tyrosine-type recombinase/integrase |
| QJ036_03783 | L | tyrosine-type recombinase/integrase |
| QJ036_03897 | L | tyrosine-type recombinase/integrase |
