# Supplementary material for: Analysis of the Taxonomy, Synteny, and Virulence Factors for Soft Rot Pathogen Pectobacterium aroidearum in Amorphophallus konjac Using Comparative Genomics
Source: Front Microbiol. 2022 Jul 13;13:868709. doi: 10.3389/fmicb.2022.868709 (PMC9326479; doi:10.3389/fmicb.2022.868709)
Supplement: Supplementary Table 6 — Type VI secretion system (T6SS) prediction for P. aroidearum genomes using web server SecReT6 v3. [file Table_6.DOCX]

| Strain ID | Sampling location | No. of T6SS cluster | No. of component proteins | No. of effectors | No. of immunity proteins | No. of accessory proteins |
| --- | --- | --- | --- | --- | --- | --- |
| QJ002 | Fuyuan,Qujing,Yunnan | 1 | 14 | 20 | 11 | 8 |
| QJ003 | Fuyuan,Qujing,Yunnan | 1 | 14 | 20 | 11 | 8 |
| QJ011 | Fuyuan,Qujing,Yunnan | 1 | 14 | 20 | 11 | 8 |
| QJ034 | Malong,Qujing,Yunnan | 1 | 14 | 16 | 7 | 8 |
| QJ036 | Malong,Qujing,Yunnan | 1 | 14 | 16 | 7 | 8 |
| QJ311 | Malong,Qujing,Yunnan | 1 | 14 | 20 | 11 | 8 |
| QJ313 | Malong,Qujing,Yunnan | 1 | 14 | 16 | 7 | 8 |
| QJ315 | Malong,Qujing,Yunnan | 1 | 14 | 16 | 7 | 8 |
| QJ316 | Malong,Qujing,Yunnan | 1 | 14 | 16 | 7 | 8 |
| AK042 | Hanbin,Ankang,Shaanxi | 1 | 15 | 21 | 10 | 8 |
| AK049 | Hanbin,Ankang,Shaanxi | 1 | 15 | 21 | 10 | 8 |

Supplementary Table 6 Type VI secretion system (T6SS) prediction for *P. aroidearum* genomes using web server SecReT6 v3

The website of SecReT6 is <https://bioinfo-mml.sjtu.edu.cn/SecReT6/index.php>. GenBank files generated by Prokka were uploaded and scanned using default parameters.
