# Supplementary material for: Analysis of the Taxonomy, Synteny, and Virulence Factors for Soft Rot Pathogen Pectobacterium aroidearum in Amorphophallus konjac Using Comparative Genomics
Source: Front Microbiol. 2022 Jul 13;13:868709. doi: 10.3389/fmicb.2022.868709 (PMC9326479; doi:10.3389/fmicb.2022.868709)
Supplement: Supplementary Table 8 — The total numbers of each gene derived from GIs. [file Table_8.DOCX]

Supplementary Table 8 The total numbers of each gene derived from GIs

| Number of times | Gene product | Gene ID |
| --- | --- | --- |
| 78 | Prophage integrase IntA | *intA* |
| 54 | Tyrosine recombinase XerC | *xerC* |
| 32 | Putative malate transporter YflS | *yflS* |
| 26 | Major exported protein | *hcpA* |
| 26 | DNA-binding transcriptional repressor YiaJ | *yiaJ* |
| 26 | Acetyl-CoA:oxalate CoA-transferase | *yfdE* |
| 25 | Prophage integrase IntS | *intS* |
| 23 | Citrate transporter | *citN* |
| 22 | Ribose import permease protein RbsC | *rbsC* |
| 19 | Tyrocidine synthase 3 | *tycC* |
| 19 | DNA topoisomerase 3 | *topB* |
| 18 | Hca operon transcriptional activator HcaR | *hcaR* |
| 17 | Toxin RTX-I translocation ATP-binding protein | *apxIB* |
| 17 | ATP-dependent zinc metalloprotease FtsH | *ftsH* |
| 16 | Vitamin B12 transporter BtuB | *btuB* |
| 16 | Tyrosine-protein kinase wzc | *wzc* |
| 16 | Glutamate racemase | *murI* |
| 16 | DNA-invertase hin | *hin* |
| 15 | Very short patch repair protein | *vsr* |
| 15 | Transposon Tn3 resolvase | *tnpR* |
| 15 | Homoserine/homoserine lactone efflux protein | *rhtB* |
| 14 | Putative O-antigen transporter | *rfbX* |
| 14 | HTH-type transcriptional activator CmpR | *cmpR* |
| 14 | Histidine-binding periplasmic protein | *hisJ* |
| 14 | GTP 3',8-cyclase | *moaA* |
| 14 | dTDP-4-amino-4,6-dideoxy-D-glucose transaminase | *vioA* |
| 14 | DNA-binding protein H-NS | *hns* |
| 14 | Cysteine/O-acetylserine efflux protein | *eamB* |
| 14 | Colicin V secretion protein CvaA | *cvaA* |
| 14 | Anaerobic nitric oxide reductase transcription regulator NorR | *norR* |
| 13 | Vitamin B12 import ATP-binding protein BtuD | *btuD* |
| 13 | UDP-glucose 6-dehydrogenase | *ugd* |
| 13 | Type-2 restriction enzyme BsuMI component YdjA | *ydjA* |
| 13 | Type I restriction enzyme EcoKI M protein | *hsdM* |
| 13 | Serine recombinase PinR | *pinR* |
| 13 | putative transport protein HsrA | *hsrA* |
| 13 | Protein TraR | *traR* |
| 13 | Modification methylase HpaII | *hpaIIM* |
| 13 | HTH-type transcriptional regulator BetI | *betI* |
| 13 | Glucose-1-phosphate thymidylyltransferase 2 | *rffH* |
| 13 | Ferric enterobactin receptor | *pfeA* |
| 13 | Bicyclomycin resistance protein | *bcr* |
| 13 | 7-cyano-7-deazaguanine synthase | *queC* |
| 13 | 2-oxoglutarate dehydrogenase E1 component | *sucA* |
| 12 | Protein-glutamate methylesterase/protein-glutamine glutaminase | *cheB* |
| 12 | Pesticin receptor | *fyuA* |
| 12 | Methyl-accepting chemotaxis protein I | *tsr* |
| 12 | Lysozyme RrrD | *rrrD* |
| 12 | Dihydrolipoyllysine-residue succinyltransferase component of 2-oxoglutarate dehydrogenase complex | *sucB* |
| 12 | Chemotaxis protein methyltransferase | *cheR* |
| 12 | Actin cross-linking toxin VgrG1 | *vgrG1* |
| 11 | Ribose import ATP-binding protein RbsA | *rbsA* |
| 11 | putative sugar kinase YdjH | *ydjH* |
| 11 | putative low molecular weight protein-tyrosine-phosphatase AmsI | *amsI* |
| 11 | Inositol 2-dehydrogenase/D-chiro-inositol 3-dehydrogenase | *iolG* |
| 11 | dTDP-glucose 4,6-dehydratase 2 | *rffG* |
| 11 | Antitoxin CcdA | *ccdA* |
| 10 | Tyrosine recombinase XerD | *xerD* |
| 10 | Toxin CcdB | *ccdB* |
| 10 | SsrA-binding protein | *smpB* |
| 10 | RutC family protein YjgH | *yjgH* |
| 10 | Reverse gyrase | *rgy* |
| 10 | putative metal-dependent hydrolase TatD | *tatD* |
| 10 | putative deoxyribonuclease RhsB | *rhsB* |
| 10 | Putative 2-hydroxyacid dehydrogenase YoaD | *yoaD* |
| 10 | Protein MtfA | *mtfA* |
| 10 | Persistence and stress-resistance toxin PasT | *pasT* |
| 10 | Persistence and stress-resistance antitoxin PasI | *pasI* |
| 10 | Pantothenate kinase | *coaA* |
| 10 | Outer membrane protein assembly factor BamE | *bamE* |
| 10 | L-xylulose/3-keto-L-gulonate kinase | *lyx* |
| 10 | Endoribonuclease SymE | *symE* |
| 10 | dTDP-4-dehydrorhamnose 3,5-epimerase | *rmlC* |
| 10 | DNA repair protein RecN | *recN* |
| 10 | DNA primase | *dnaG* |
| 10 | Bifunctional ligase/repressor BirA | *birA* |
| 10 | ATP-dependent DNA helicase Rep | *rep* |
| 10 | Acetyltransferase | *ttr* |
| 9 | UDP-N-acetylenolpyruvoylglucosamine reductase | *murB* |
| 9 | Toxin YkfI | *ykfI* |
| 9 | Protein DsrB | *dsrB* |
| 9 | NAD kinase | *nadK* |
| 9 | dTDP-4-dehydrorhamnose reductase | *rmlD* |
| 9 | Dihydrolipoyl dehydrogenase | *lpdG* |
| 9 | Diguanylate cyclase DosC | *dosC* |
| 9 | Antitoxin YfjZ | *yfjZ* |
| 9 | 3-oxoacyl-[acyl-carrier-protein] reductase FabG | *fabG* |
| 8 | UDP-galactopyranose mutase | *glf* |
| 8 | Immunity protein CdiI-o11 | *cdiI4* |
| 8 | GTPase Era | *era* |
| 8 | Gluconate 5-dehydrogenase | *gno* |
| 8 | DNA primase TraC | *traC* |
| 8 | Antitoxin VapB | *vapB* |
| 8 | 50S ribosomal protein L16 3-hydroxylase | *roxA* |
| 7 | Unsaturated chondroitin disaccharide hydrolase | *ugl* |
| 7 | tRNA(fMet)-specific endonuclease VapC | *vapC* |
| 7 | Sugar phosphatase YidA | *yidA* |
| 7 | Single-stranded DNA-binding protein | *ssb* |
| 7 | Replicative DNA helicase | *dnaB* |
| 7 | Putative defective protein IntQ | *intQ* |
| 7 | PTS system mannose-specific EIID component | *manZ* |
| 7 | Polyketide synthase PksJ | *pksJ* |
| 7 | N-acetylgalactosamine permease IIC component 1 | *agaC* |
| 7 | Flagellin | *fliC* |
| 7 | ABC transporter periplasmic-binding protein YtfQ | *ytfQ* |
| 6 | UDP-4-amino-4-deoxy-L-arabinose--oxoglutarate aminotransferase | *arnB* |
| 6 | Trans-acting regulatory protein HvrA | *hvrA* |
| 6 | S-methyl-5'-thioadenosine phosphorylase | *mtnP* |
| 6 | Ribosomal protein S12 methylthiotransferase RimO | *rimO* |
| 6 | Respiratory nitrate reductase 1 gamma chain | *narI* |
| 6 | Putative nuclease YbcO | *ybcO* |
| 6 | Phosphoglycolate phosphatase | *gph* |
| 6 | Multidrug resistance protein MdtC | *mdtC* |
| 6 | HTH-type transcriptional regulator PgrR | *pgrR* |
| 6 | HTH-type transcriptional regulator DmlR | *dmlR* |
| 6 | HTH-type transcriptional activator RhaR | *rhaR* |
| 6 | GDP-mannose pyrophosphatase NudK | *nudK* |
| 6 | DNA adenine methylase | *dam* |
| 5 | UTP--glucose-1-phosphate uridylyltransferase | *galF* |
| 5 | Putative deoxyribonuclease RhsC | *rhsC* |
| 5 | Methyl-accepting chemotaxis protein III | *trg* |
| 5 | Membrane-bound lytic murein transglycosylase F | *mltF* |
| 5 | Leucine-responsive regulatory protein | *lrp* |
| 5 | HTH-type transcriptional regulator SutR | *sutR* |
| 5 | HTH-type transcriptional regulator GltC | *gltC* |
| 5 | HTH-type transcriptional regulator AscG | *ascG* |
| 5 | GMP synthase [glutamine-hydrolyzing] | *guaA* |
| 5 | GDP-perosamine synthase | *rfbE* |
| 5 | GDP-perosamine N-acetyltransferase | *perB* |
| 5 | Endonuclease NucS | *nucS* |
| 5 | DNA polymerase III subunit theta | *holE* |
| 5 | CDP-diacylglycerol--glycerol-3-phosphate 3-phosphatidyltransferase | *pgsA* |
| 5 | Acetylornithine aminotransferase | *argD* |
| 5 | 6-phosphogluconate dehydrogenase, decarboxylating | *gnd* |
| 4 | UvrABC system protein C | *uvrC* |
| 4 | Transcription termination/antitermination protein NusG | *nusG* |
| 4 | Secretory immunoglobulin A-binding protein EsiB | *esiB* |
| 4 | scyllo-inositol 2-dehydrogenase (NADP(+)) IolU | *iolU* |
| 4 | putative protein adenylyltransferase Fic | *fic* |
| 4 | Putative amino-acid ABC transporter-binding protein YhdW | *yhdW* |
| 4 | Protein translocase subunit SecE | *secE* |
| 4 | Protein GrpE | *grpE* |
| 4 | Lactose permease | *lacY* |
| 4 | Inner membrane amino-acid ABC transporter permease protein YhdY | *yhdY* |
| 4 | HTH-type transcriptional regulator NimR | *nimR* |
| 4 | Glutathione-regulated potassium-efflux system ancillary protein KefF | *kefF* |
| 4 | General stress protein A | *gspA* |
| 4 | Fosfomycin resistance protein AbaF | *abaF* |
| 4 | Elongation factor Tu 2 | *tufB* |
| 4 | DNA-directed RNA polymerase subunit beta' | *rpoC* |
| 4 | DNA-directed RNA polymerase subunit beta | *rpoB* |
| 4 | Dimodular nonribosomal peptide synthase | *dhbF* |
| 4 | Cation/acetate symporter ActP | *actP* |
| 4 | Apulose-4-phosphate transketolase subunit B | *aptB* |
| 4 | Apulose-4-phosphate transketolase subunit A | *aptA* |
| 4 | 5-amino-6-(5-phospho-D-ribitylamino)uracil phosphatase YbjI | *ybjI* |
| 4 | 50S ribosomal protein L7/L12 | *rplL* |
| 4 | 50S ribosomal protein L11 | *rplK* |
| 4 | 50S ribosomal protein L10 | *rplJ* |
| 4 | 50S ribosomal protein L1 | *rplA* |
| 4 | 2-succinylbenzoate--CoA ligase | *menE* |
| 3 | UDP-N-acetyl-D-glucosamine 6-dehydrogenase | *wbpA* |
| 3 | Type IV secretion system protein virB9 | *virB9* |
| 3 | Type IV secretion system protein virB4 | *virB4* |
| 3 | Type IV secretion system protein PtlE | *ptlE* |
| 3 | RNA pyrophosphohydrolase | *rppH* |
| 3 | Right origin-binding protein | *rob* |
| 3 | Response regulator UvrY | *uvrY* |
| 3 | RecBCD enzyme subunit RecB | *recB* |
| 3 | putative deoxyribonuclease RhsA | *rhsA* |
| 3 | Phosphoribosyl 1,2-cyclic phosphate phosphodiesterase | *phnP* |
| 3 | Peptidoglycan D,D-transpeptidase FtsI | *ftsI* |
| 3 | Molybdenum transport system permease protein ModB | *modB* |
| 3 | HTH-type transcriptional regulator CatM | *catM* |
| 3 | D-serine/D-alanine/glycine transporter | *cycA* |
| 3 | Anti-adapter protein IraP | *iraP* |
| 3 | Adenosylmethionine-8-amino-7-oxononanoate aminotransferase | *bioA* |
| 2 | UvrABC system protein B | *uvrB* |
| 2 | UvrABC system protein A | *uvrA* |
| 2 | UDP-N-acetylglucosamine 4-epimerase | *wbgU* |
| 2 | tRNA-cytidine(32) 2-sulfurtransferase | *ttcA* |
| 2 | Thymidylate synthase | *thyA* |
| 2 | sugar efflux transporter | *sotB* |
| 2 | RNA polymerase sigma factor FliA | *fliA* |
| 2 | RNA chaperone ProQ | *proQ* |
| 2 | Rhamnosyltransferase WbbL | *wbbL* |
| 2 | Prophage tail fiber assembly protein TfaE | *tfaE* |
| 2 | Phosphomannomutase/phosphoglucomutase | *algC* |
| 2 | Peptidoglycan hydrolase FlgJ | *flgJ* |
| 2 | PCP degradation transcriptional activation protein | *pcpR* |
| 2 | Oligoribonuclease | *orn* |
| 2 | Multidrug resistance protein MdtA | *mdtA* |
| 2 | Mrr restriction system protein | *mrr* |
| 2 | Modification methylase BspRI | *bspRIM* |
| 2 | Metal-pseudopaline receptor CntO | *cntO* |
| 2 | LexA repressor | *lexA* |
| 2 | Inner membrane protein YfdC | *yfdC* |
| 2 | HTH-type transcriptional regulator VirS | *virS* |
| 2 | HTH-type transcriptional regulator HexR | *hexR* |
| 2 | Hemolysin transporter protein ShlB | *shlB* |
| 2 | GTPase Der | *der* |
| 2 | Flagellum-specific ATP synthase | *fliI* |
| 2 | Flagellin 2 | *fliC2* |
| 2 | Flagellar secretion chaperone FliS | *fliS* |
| 2 | Flagellar protein FliT | *fliT* |
| 2 | Flagellar protein FliO | *fliO* |
| 2 | Flagellar P-ring protein | *flgI* |
| 2 | Flagellar M-ring protein | *fliF* |
| 2 | Flagellar motor switch protein FliN | *fliN* |
| 2 | Flagellar motor switch protein FliM | *fliM* |
| 2 | Flagellar motor switch protein FliG | *fliG* |
| 2 | Flagellar L-ring protein | *flgH* |
| 2 | Flagellar hook-basal body complex protein FliE | *fliE* |
| 2 | Flagellar hook-associated protein 3 | *flgL* |
| 2 | Flagellar hook-associated protein 2 | *fliD* |
| 2 | Flagellar hook-associated protein 1 | *flgK* |
| 2 | Flagellar hook protein FlgE | *flgE* |
| 2 | Flagellar FliJ protein | *fliJ* |
| 2 | Flagellar biosynthetic protein FliR | *fliR* |
| 2 | Flagellar biosynthetic protein FliQ | *fliQ* |
| 2 | Flagellar biosynthetic protein FliP | *fliP* |
| 2 | Flagellar basal-body rod protein FlgG | *flgG* |
| 2 | Flagellar basal-body rod protein FlgF | *flgF* |
| 2 | Flagellar basal-body rod protein FlgC | *flgC* |
| 2 | Flagellar assembly protein FliH | *fliH* |
| 2 | D-inositol-3-phosphate glycosyltransferase | *mshA* |
| 2 | Cytosine permease | *codB* |
| 2 | CRISPR system Cms endoribonuclease Csm3 | *csm3* |
| 2 | Chaperone protein HtpG | *htpG* |
| 2 | Bifunctional transcriptional activator/DNA repair enzyme AdaA | *adaA* |
| 2 | Basal-body rod modification protein FlgD | *flgD* |
| 2 | Adenylate cyclase CyaB | *cyaB* |
| 2 | 3-hydroxybenzoate 4-monooxygenase | *mobA* |
| 2 | 3-carboxy-cis,cis-muconate cycloisomerase | *pcaB* |
| 2 | 2-succinyl-6-hydroxy-2, 4-cyclohexadiene-1-carboxylate synthase | *menH* |
| 2 | 2,3,4,5-tetrahydropyridine-2,6-dicarboxylate N-acetyltransferase | *dapH* |
| 1 | Undecaprenyl-phosphate 4-deoxy-4-formamido-L-arabinose transferase | *arnC* |
| 1 | UDP-glucose:undecaprenyl-phosphate glucose-1-phosphate transferase | *wcaJ* |
| 1 | Ubiquinone biosynthesis O-methyltransferase, mitochondrial | *COQ3* |
| 1 | Trigger factor | *tig* |
| 1 | Toxin HigB-1 | *higB-1* |
| 1 | Threonylcarbamoyl-AMP synthase | *tsaC* |
| 1 | Threonine/homoserine exporter RhtA | *rhtA* |
| 1 | Thiol:disulfide interchange protein DsbD | *dsbD* |
| 1 | TDP-4-oxo-6-deoxy-alpha-D-glucose-3, 4-oxoisomerase | *fdtA* |
| 1 | Small ribosomal subunit biogenesis GTPase RsgA | *rsgA* |
| 1 | Shikimate dehydrogenase (NADP(+)) | *aroE* |
| 1 | Ribose 1,5-bisphosphate phosphokinase PhnN | *phnN* |
| 1 | Riboflavin transporter RfnT | *rfnT* |
| 1 | RCS-specific HTH-type transcriptional activator RclR | *rclR* |
| 1 | putative protein YybH | *yybH* |
| 1 | Putative prophage major tail sheath protein | *gpFI* |
| 1 | Putative phosphonates utilization ATP-binding protein PhnK | *phnK* |
| 1 | putative HTH-type transcriptional regulator YbaQ | *ybaQ* |
| 1 | putative FMN/FAD exporter YeeO | *yeeO* |
| 1 | putative ABC transporter ATP-binding protein YknY | *yknY* |
| 1 | PTS system N,N'-diacetylchitobiose-specific EIIA component | *chbA* |
| 1 | Protoporphyrinogen IX dehydrogenase [menaquinone] | *hemG* |
| 1 | Protoheme IX farnesyltransferase | *cyoE* |
| 1 | Protein YrdA | *yrdA* |
| 1 | Protein Smg | *smg* |
| 1 | Primosomal protein 1 | *dnaT* |
| 1 | Phosphatidylserine decarboxylase proenzyme | *psd* |
| 1 | Peptidyl-lysine N-acetyltransferase YjaB | *yjaB* |
| 1 | Outer membrane protein X | *ompX* |
| 1 | N-ethylmaleimide reductase | *nemA* |
| 1 | mRNA interferase toxin RelE | *relE* |
| 1 | Motility protein B | *motB* |
| 1 | Motility protein A | *motA* |
| 1 | Mannose-1-phosphate guanylyltransferase RfbM | *rfbM* |
| 1 | Mannose-1-phosphate guanylyltransferase 1 | *manC1* |
| 1 | Macrolide export ATP-binding/permease protein MacB | *macB* |
| 1 | Low specificity L-threonine aldolase | *ltaE* |
| 1 | Lon protease | *lon* |
| 1 | Lipid III flippase | *wzxE* |
| 1 | lipid II flippase MurJ | *murJ* |
| 1 | Inner membrane transport protein YajR | *yajR* |
| 1 | Inner membrane protein YgaP | *ygaP* |
| 1 | HTH-type transcriptional regulator PerR | *perR* |
| 1 | HTH-type transcriptional regulator BenM | *benM* |
| 1 | Glutathione-regulated potassium-efflux system ancillary protein KefG | *kefG* |
| 1 | Glutathione hydrolase proenzyme | *ggt* |
| 1 | Glucose-1-phosphate cytidylyltransferase | *rfbF* |
| 1 | GDP-mannose mannosyl hydrolase | *gmm* |
| 1 | GDP-L-fucose synthase | *fcl* |
| 1 | Gamma-glutamyl phosphate reductase | *proA* |
| 1 | Flagellar transcriptional regulator FlhD | *flhD* |
| 1 | Flagellar transcriptional regulator FlhC | *flhC* |
| 1 | dTDP-3-amino-3,6-dideoxy-alpha-D-galactopyranose transaminase | *fdtB* |
| 1 | DNA-binding transcriptional regulator BolA | *bolA* |
| 1 | DNA-binding protein HU-beta | *hupB* |
| 1 | DNA topoisomerase 1 | *topA* |
| 1 | DNA gyrase inhibitor | *sbmC* |
| 1 | Divalent-cation tolerance protein CutA | *cutA* |
| 1 | D-alanine--D-alanyl carrier protein ligase | *dltA* |
| 1 | Cytochrome bo(3) ubiquinol oxidase subunit 4 | *cyoD* |
| 1 | Cytochrome bo(3) ubiquinol oxidase subunit 3 | *cyoC* |
| 1 | Cytochrome bo(3) ubiquinol oxidase subunit 2 | *cyoA* |
| 1 | Cytochrome bo(3) ubiquinol oxidase subunit 1 | *cyoB* |
| 1 | Cryptic outer membrane porin BglH | *bglH* |
| 1 | CRISPR-associated endonuclease/helicase Cas3 | *ygcB* |
| 1 | CDP-abequose synthase | *rfbJ* |
| 1 | CDP-6-deoxy-L-threo-D-glycero-4-hexulose-3- dehydrase reductase | *ascD* |
| 1 | Bacterial non-heme ferritin | *ftnA* |
| 1 | ATP-dependent RNA helicase DbpA | *dbpA* |
| 1 | ATP-dependent Clp protease proteolytic subunit | *clpP* |
| 1 | ATP-dependent Clp protease ATP-binding subunit ClpX | *clpX* |
| 1 | Aryl-phospho-beta-D-glucosidase BglH | *bglH* |
| 1 | Anhydromuropeptide permease | *ampG* |
| 1 | Anaerobic sulfatase-maturating enzyme | *chuR* |
| 1 | Alpha-D-ribose 1-methylphosphonate 5-triphosphate synthase subunit PhnL | *phnL* |
| 1 | Alpha-D-ribose 1-methylphosphonate 5-triphosphate diphosphatase | *phnM* |
| 1 | Alpha-D-ribose 1-methylphosphonate 5-phosphate C-P lyase | *phnJ* |
| 1 | Acrylyl-CoA reductase AcuI | *acuI* |
| 1 | 2-nitroimidazole transporter | *nimT* |
